# Biopolymer—Nanoparticle Interactions in 3D-Printing for Biomedical Applications: Advantages, Limitations and Future Perspectives

**DOI:** 10.3390/polym18091038

**Published:** 2026-04-24

**Authors:** Miguel Muñoz-Silva, Rafaela García-Álvarez, Elena Pérez, Carla Jiménez-Jiménez, Adrián Esteban-Arranz

**Affiliations:** 1 Nanocaging Research Group, Department of Pharmacy and Nutrition, Faculty of Biomedical and Health Sciences, Universidad Europea de Madrid, Campus de Villaviciosa, Calle Tajo s/n, 28670 Villaviciosa de Odón, Madrid, Spain; miguel.munoz@universidadeuropea.es (M.M.-S.); rafaela.garcia@universidadeuropea.es (R.G.-Á.); 2 Nanocaging Research Group, Department of Biosciences, Faculty of Biomedical and Health Sciences, Universidad Europea de Madrid, Campus de Villaviciosa, Calle Tajo s/n, 28670 Villaviciosa de Odón, Madrid, Spain; elena.perez2@universidadeuropea.es (E.P.); carla.jimenez@universidadeuropea.es (C.J.-J.)

**Keywords:** nanoparticles, biopolymers, interactions, nanocomposites, 3D-printing, biomedical applications, AI-driven materials

## Abstract

This review comprehensively examines the incorporation of nanoparticles (NPs) into biopolymers for 3D printing in biomedical applications, integrating material design, processing strategies, and translational considerations within a unified framework. Different types of NPs are analyzed regarding their effects on mechanical reinforcement, rheological modulation, and structural organization of biopolymeric matrices. The discussion covers principal additive manufacturing technologies, including extrusion-based systems such as fused deposition modeling (FDM) and direct ink writing (DIW), vat photopolymerization, powder-bed fusion (SLS), and emerging in situ nanoparticle formation approaches, emphasizing how nanoparticle loading and surface functionalization govern yield stress, shear-thinning behavior, viscoelastic recovery, and dimensional fidelity while mitigating agglomeration and optimizing interfacial interactions. Comparative evaluation of compressive modulus, strength, toughness, crystallinity, and porosity establishes structure–property–processing relationships directly linked to printability and functional performance. Biomedical applications are addressed in tissue engineering, biosensing, controlled and targeted drug delivery, and bioimaging, highlighting the balance between bioactivity and manufacturability. Finally, critical challenges—including compatibility, reproducibility, biological safety, long-term stability, regulatory adaptation, and environmental impact—are discussed, alongside future perspectives focused on green nanomaterials, AI-driven predictive formulation design, and digital twins for real-time monitoring and quality control in nano-enabled additive manufacturing.

## 1. Theoretical Background—Introduction

3D printing has become a key processing strategy for bio-based polymers in biomedical applications due to its versatility, scalability, and material adaptability. Despite these advantages, many bio-derived matrices exhibit intrinsic limitations, including insufficient mechanical strength, limited structural stability after deposition, and restricted functional responsiveness when processed under extrusion conditions [1,2]. These constraints highlight the need for material design strategies capable of simultaneously enhancing performance and preserving printability. Among the most widely explored approaches is the incorporation of nanoscale fillers to tailor structural and functional properties within bio-based matrices [1].

In biomedical contexts, these material limitations directly affect scaffold integrity, pore architecture preservation, and the long-term stability of patient-specific constructs, thereby reinforcing the need for rational nanocomposite design strategies tailored to fabrication.

Therefore, the aim is to provide a critical overview of the existing literature and to identify general trends in the influence of nanoparticle incorporation in polymer matrices for additive manufacturing.

### 1.1. Effect of Nanoparticle Incorporation into Biopolymers for 3D Printing

The addition of nanoparticles into bio-based polymers for 3D printing has been widely reported as an effective strategy to enhance mechanical performance and introduce additional functionality in materials intended for biomedical applications [1,3].

In this framework, “synergistic effects” refer not merely to additive improvements in isolated properties but to coupled structure–property–processing interactions arising from nanoscale interfacial phenomena and particle network formation. At the nanoscale, nanoparticles can interact with polymer chains and influence internal reactions such as crystallization behavior in semi-crystalline thermoplastics. Specifically, nanoparticle incorporation has been shown to act as heterogeneous nucleation sites in PLA-based systems, modifying crystallization kinetics and affecting thermal and dimensional behavior during printing [4].

Synergistic reinforcement in polymer nanocomposites is commonly associated with efficient stress transfer at the polymer–nanoparticle interface, particularly when dispersion is homogeneous [1,5]. In high-aspect-ratio systems such as carbon nanotube-based composites, electrical percolation can be achieved at relatively low filler contents, enabling the formation of conductive networks [6]. Nevertheless, percolation is typically accompanied by marked increases in viscoelastic properties, reflecting the formation of particle networks within the matrix [5,6]. Such transitions can significantly influence extrusion pressure requirements and flow behavior.

Taken together, the literature indicates that nanoparticle incorporation into bio-based matrices for extrusion-based biomedical fabrication represents a balance between enhanced mechanical or functional performance and the rheological and processing constraints introduced by filler–matrix interactions [1,7]. The extent of this balance depends strongly on nanoparticle chemistry, morphology, dispersion quality, and concentration, factors that ultimately determine printability, structural fidelity, and final performance in extrusion-based systems. This balance between reinforcement and processability ultimately defines the advantages and limitations associated with different nanoparticle types when incorporated into bio-based materials for extrusion-based 3D printing in biomedical applications.

Therefore, nanoparticle incorporation in extrusion-based biofabrication should be evaluated through an integrated structure–property–processing perspective, where reinforcement efficiency, rheological modification, extrusion stability, and biological safety are considered simultaneously rather than independently.

#### 1.1.1. Metallic Nanoparticles

Within this structure–property–processing framework, different nanoparticle families introduce distinct reinforcement mechanisms, functional contributions, and processing constraints. Metallic nanoparticles are incorporated into bio-based extrusion systems primarily to introduce antimicrobial, magnetic, conductive, or photothermal functionality in biomedical constructs [8,9]. In contrast to ceramic nanofillers, which mainly contribute to structural reinforcement, metallic nanoparticles are typically selected for their functional activity, enabling bioactive, responsive, or multifunctional printed scaffolds.

From a structural standpoint, metallic nanoparticles generally exhibit limited intrinsic reinforcement efficiency compared to high-aspect-ratio or stiff ceramic nanofillers, as their typically isotropic morphology restricts effective stress transfer within polymer matrices. Consequently, their incorporation is predominantly driven by functional requirements rather than by mechanical optimization objectives [5].

Silver nanoparticles (AgNPs) are among the most extensively studied metallic nanomaterials due to their broad-spectrum antimicrobial activity, which is mainly attributed to ion release and oxidative stress-related mechanisms, including the generation of reactive oxygen species and membrane disruption [9]. When integrated into extrusion-printed biodegradable scaffolds, AgNPs can significantly reduce bacterial colonization, representing a major advantage for implantable constructs and infection-prone biomedical devices [10]. In extrusion-based systems, however, antimicrobial efficacy is strongly concentration-dependent. Increasing AgNP loading may enhance antibacterial performance but simultaneously influence rheological behavior by elevating zero-shear viscosity and modifying viscoelastic response, potentially narrowing the processing window required for stable filament deposition [5,7].

Iron oxide nanoparticles (Fe_3_O_4_) introduce magnetic responsiveness, enabling remotely actuated or magnetically guided biomedical systems such as stimuli-responsive scaffolds or magnetically assisted drug delivery constructs [8]. In extrusion-based fabrication, magnetic particles allow the representation of externally controllable architectures, which represents a clear functional advantage. Nevertheless, their higher density relative to polymer matrices may pose challenges in low-viscosity inks or hydrogels, where maintaining homogeneous particle distribution during prolonged processing requires careful rheological tuning.

Zinc oxide (ZnO) and copper oxide (CuO) nanoparticles provide antimicrobial activity through mechanisms including reactive oxygen species generation and membrane disruption [11]. Their integration into bio-based polymers for extrusion printing expands the range of antibacterial functionalities beyond silver-based systems. However, metallic and metal oxide nanoparticles typically exhibit high surface energy and a pronounced tendency toward aggregation if not adequately surface-modified.

Gold nanoparticles (AuNPs), although less frequently used for mechanical reinforcement, are incorporated when photothermal, sensing, or imaging capabilities are required [12]. Their high surface tunability and biocompatibility enable multifunctional constructs at relatively low filler loadings. However, particle size, surface chemistry, and concentration can still influence composite rheology and interfacial interactions within the polymer matrix [5]. As with other metallic nanoparticles, achieving homogeneous dispersion remains essential to prevent local stress concentrations and ensure reproducible extrusion behavior. This functional dominance over structural reinforcement differentiates metallic nanoparticles from other nanofiller classes and underscores their role as bioactivity enhancers rather than primary load-bearing reinforcements in extrusion-printed composites. In this sense, metallic nanoparticles are rarely employed as primary structural reinforcements in load-bearing biofabricated constructs but rather as multifunctional modifiers integrated under strict biological and rheological constraints.

Overall, metallic nanoparticles provide significant functional advantages in bio-based extrusion systems for biomedical applications, particularly in terms of antimicrobial activity, magnetic responsiveness, and photothermal or sensing capabilities. However, their incorporation is accompanied by limitations related to dispersion stability, viscosity increase, aggregation tendency, and dose-dependent cytotoxicity. From a processing perspective, the balance between functional enhancement and extrusion stability is governed by particle size, surface modification, concentration, and interaction with the surrounding bio-based matrix. Careful optimization of these parameters is therefore essential to ensure that multifunctionality does not compromise printability, structural fidelity, or biological safety [7,9].

#### 1.1.2. Ceramic and Inorganic Nanoparticles

Ceramic and inorganic nanoparticles such as nano-hydroxyapatite (nHA), bioactive glass (BG), and silica are extensively incorporated into bio-based polymers to enhance stiffness, bioactivity, and thermal stability in extrusion-based biomedical systems [13,14]. In contrast to metallic nanoparticles, their primary contribution lies in structural reinforcement and osteoconductive potential. Unlike metallic nanoparticles, whose incorporation is often function-driven, ceramic nanofillers are predominantly selected to enhance load-bearing capacity, interfacial stiffness, and dimensional stability within extrusion-printed bio-based systems, thereby positioning them as structural reinforcements rather than multifunctional additives.

Nano-hydroxyapatite (nHA), due to its compositional similarity to the mineral phase of bone, exhibits excellent biocompatibility and intrinsic osteoconductivity [13].

Bioactive glass (BG) nanoparticles introduce additional functionality through ionic dissolution behavior. Upon exposure to physiological environments, BG releases ions such as Ca^2+^ and Si^4+^, which promote hydroxyapatite layer formation and stimulate osteogenic responses [14,15]. In polymer composites processed by extrusion, this dissolution-driven bioactivity represents a significant advantage for bone-regenerative applications. However, ion release kinetics may locally modify pH and influence degradation rates of surrounding bio-based polymers, particularly in hydrolytically sensitive systems [15]. Therefore, compositional design and filler loading must be carefully optimized.

The intrinsic rigidity of ceramic particles restricts polymer chain mobility at high volume fractions, which may result in increased brittleness and reduced fracture toughness [16]. The balance between stiffness enhancement and toughness preservation is therefore a critical consideration in the design of printable bio-based nanocomposites.

Overall, ceramic and inorganic nanoparticles provide clear advantages in terms of mechanical reinforcement and bioactivity in extrusion-based biomedical fabrication. Nevertheless, their incorporation narrows the printable window due to viscosity increase, interfacial incompatibility, and the potential transition toward brittle mechanical behavior at elevated loadings [7,16].

#### 1.1.3. Carbon-Based Nanomaterials

Carbon-based nanomaterials such as carbon nanotubes (CNTs), graphene, graphene oxide (GO), and reduced graphene oxide (rGO) exhibit high intrinsic mechanical properties and electrical conductivity, making them attractive for multifunctional reinforcement in bio-based extrusion systems intended for biomedical applications [17,18,19]. Unlike ceramic fillers, which primarily increase stiffness and bioactivity, carbon-based nanofillers can provide a coupled improvement in mechanical performance and electrical functionality, which is particularly relevant for electroactive constructs and conductive biomaterials [17].

In contrast to ceramic fillers, whose reinforcement derives primarily from stiffness contrast, carbon-based nanomaterials modify composite performance through percolation-driven network formation phenomena that simultaneously influence mechanical response, electrical conductivity, and viscoelastic behavior [18].

A major advantage of CNTs and graphene derivatives is their ability to form percolation networks at relatively low filler concentrations due to their high aspect ratio and large specific surface area. Once percolation is achieved, conductive pathways can be established within otherwise insulating polymer matrices, enabling electrical conductivity without requiring high overall filler contents [6,18]. In practice, the percolation threshold and the stability of conductive networks are strongly dependent on dispersion quality and processing history, which becomes especially critical in extrusion-based fabrication, where shear history and residence time can alter microstructure development [5].

Overall, carbon-based nanomaterials offer clear advantages in bio-based extrusion systems by enabling simultaneous mechanical reinforcement and electrical functionality at relatively low filler contents [17,18,19]. However, these benefits are counterbalanced by limitations related to dispersion and agglomeration, abrupt rheological transitions near percolation, potential constraints on interlayer bonding, and dispersion-dependent biological response [5,6,20]. Achieving an optimal balance between conductivity, reinforcement, and extrusion stability therefore requires careful control of concentration, dispersion strategy, and surface chemistry [5,18].

Beyond sp^2^-dominated carbon nanostructures such as CNTs and graphene derivatives, other carbon allotropes with distinct structural and surface characteristics have also been explored for extrusion-based biomedical systems.

#### 1.1.4. Nanodiamonds and Diamond-Based Nanomaterials

Nanodiamonds (NDs) and diamond-based nanomaterials represent a distinct subclass of carbon nanostructures characterized by sp^3^-hybridized carbon cores, high hardness, chemical stability, and surface-rich functional groups [21,22]. In contrast to graphene and carbon nanotubes, whose properties are dominated by sp^2^-conjugated structures and electrical conductivity, nanodiamonds exhibit excellent mechanical robustness, tunable surface chemistry, and high biocompatibility, making them increasingly attractive for biomedical applications in extrusion-based bio-based systems [21,23].

Positioned between conductive sp^2^ carbon nanostructures and rigid ceramic fillers, nanodiamonds occupy a distinct design space characterized by surface-mediated interfacial reinforcement without reliance on electrical percolation mechanisms. Their effectiveness, therefore, relies less on network formation phenomena and more on interfacial engineering strategies tailored to the specific polymeric environment.

Beyond mechanical reinforcement, nanodiamonds have attracted considerable attention for biomedical applications due to their reported low cytotoxicity and potential as drug delivery platforms through surface functionalization [23,24]. Their chemical stability and resistance to oxidative degradation distinguish them from other carbon-based nanomaterials, potentially offering advantages in long-term implantable or load-bearing constructs. Nevertheless, biological response remains dependent on particle size, aggregation state, and surface chemistry. In extrusion-printed composites, inhomogeneous dispersion may lead to localized stiffness variations and heterogeneous functional performance.

Overall, nanodiamonds and diamond-based nanomaterials offer a unique combination of mechanical reinforcement potential, surface tunability, and favorable biocompatibility in bio-based extrusion systems for biomedical applications. However, as with other nanoparticle types, their successful incorporation depends on achieving an optimal balance between interfacial enhancement, rheological control, dispersion stability, and printability. This balance ultimately determines whether the intrinsic advantages of nanodiamonds translate into improved structural performance without compromising extrusion process stability or biological safety [22,23].

Collectively, the incorporation of distinct nanoparticle classes into biopolymer matrices for biomedical applications reveals differentiated structure–property–processing paradigms. Metallic nanoparticles primarily introduce bioactive or responsive functionality under strict dose-dependent biological constraints and limited structural reinforcement efficiency. Ceramic and inorganic fillers predominantly enhance structural stiffness and osteoconductivity, with increased viscosity and potential brittleness at elevated loadings. Carbon-based nanomaterials enable coupled mechanical–electrical performance through percolation-driven network formation but exhibit pronounced rheological sensitivity and dispersion challenges near the percolation threshold. Nanodiamonds provide surface-mediated interfacial reinforcement with comparatively moderate electrical activity yet remain susceptible to aggregation-driven limitations.

### 1.2. Additive Manufacturing Technologies for 3D-Printing

Additive manufacturing technologies play a decisive role in determining the structural performance, functional properties, and biomedical applicability and potential of nanoparticle-reinforced bio-based materials. The incorporation of nanomaterials into polymeric matrices introduces additional constraints related to dispersion, rheology, thermal stability, and process compatibility, making the choice of printing technique a critical design decision rather than a purely technical one. This section provides an overview of the main 3D printing technologies applied to nanocomposite biomaterials, with emphasis on their working principles, advantages, and limitations in biomedical contexts.

#### 1.2.1. Extrusion-Based Technologies: Fused Deposition Modeling (FDM) and Direct Ink Writing (DIW)

Extrusion-based techniques are among the most widely used approaches for processing bio-based polymers and nanocomposites due to their relative simplicity, material versatility, and stability. In these methods, material is deposited layer-by-layer through a nozzle under thermal or mechanical driving forces. However, the presence of nanoparticles strongly influences melt flow behavior or ink rheology, interlayer adhesion, and printing resolution and fidelity [25]. As a result, careful formulation and process optimization are needed.

Fused deposition modeling (FDM) is one of the most established extrusion-based techniques and is commonly used with thermoplastic bio-based polymers such as polylactic acid (PLA) and polycaprolactone (PCL) [26]. In FDM, a solid filament is melted and extruded through a heated nozzle, followed by solidification upon deposition.

The incorporation of nanoparticles into FDM filaments has been widely explored to enhance mechanical strength, thermal stability, bioactivity, and antimicrobial performance. Low nanoparticle contents (typically 1–2 wt.% for many systems) often give the best balance between reinforcement and printability, improving tensile strength and modulus [27]. For example, bioactive glass and hydroxyapatite nanoparticles have been introduced to improve osteoconductivity, while carbon-based and metallic nanomaterials have been explored to confer electrical conductivity [28] or antibacterial functionality [1].

However, nanoparticle addition typically increases melt viscosity and modifies crystallization behavior [29], which may compromise flow stability, interlayer diffusion, and bonding quality [26]. Excessive loading or poor dispersion can generate agglomerates acting as stress concentration sites, ultimately reducing mechanical performance rather than reinforcing it [30]. In this study, PLA/Cu nanoparticle filaments improve thermal stability, crystallization, and tensile strength, but mechanical behavior is highly sensitive to dispersion quality and interfacial cohesion [31]. Furthermore, high processing temperatures limit the incorporation of thermally sensitive biomolecules or living cells. For these reasons, FDM-based nanocomposites are primarily suited for acellular scaffolds, structural implants, and drug-loaded systems that allow post-printing functionalization.

Direct Ink Writing (DIW) represents a low-temperature extrusion-based technique particularly well suited for bio-based hydrogels, polymer solutions, and cell-laden bioinks used in tissue engineering and regenerative medicine [32,33]. In DIW, viscoelastic inks are extruded under controlled pressure, requiring a finely tuned rheological profile to ensure both printability and shape retention. For nanocomposite bioinks, nanoparticles can be incorporated to modulate mechanical reinforcement, bioactivity, electrical conductivity, or controlled release behavior [34]. However, successful printing depends on achieving shear-thinning behavior, sufficient yield stress to maintain structural integrity after deposition, and rapid structural recovery [33,35]. Nanoparticle agglomeration or excessive concentration can disrupt flow behavior, induce nozzle clogging, and compromise printing resolution. Thus, in DIW systems, the central challenge lies in balancing functional enhancement with rheological stability, particularly when cytocompatibility and cell viability must be preserved.

The printability of extrusion-based processes can be rationalized in terms of a rheological processing window, defined by the balance between flow through the nozzle and shape retention after deposition. This is defined by stress, viscosity, and elasticity required to prevent filament spreading or collapse, and the viscosity and pressure above which extrusion becomes unstable or unfeasible [36,37].

In direct ink writing (DIW), printability and shape fidelity are governed by yield-stress fluids with pronounced shear-thinning behavior. DIW inks must yield under the applied nozzle pressure, flow readily during extrusion, and recover sufficient yield stress after deposition to maintain structural integrity [38,39]. In bioprinting and soft hydrogel-based DIW, these requirements shift toward yield stress values to preserve cell viability and homogenous cell distribution, which can be hindered and impaired with high yield stress [40,41].

In contrast, fused deposition modeling (FDM) relies on the flow of yield-free polymer melts, where printability is governed by a viscosity window that balances extrusion, filament stability, and interlayer adhesion. The melted viscosity must be low enough to enable continuous flow, yet sufficiently high to preserve filament shape after deposition. Therefore, optimal rheological windows are not universal but depend on the specific ink formulation and application requirements [42].

#### 1.2.2. Vat Photopolymerization Technologies

Stereolithography (SLA), digital light processing (DLP), and two-photon polymerization (2PP) are vat photopolymerization technologies. These rely on spatially controlled light-induced curing of liquid photopolymer resins. All these methods offer high printing resolution and smooth surface finishes, making them particularly attractive for fabricating intricate biomedical micro-architecture [43,44].

The incorporation of nanoparticles into photocurable resins has been explored to enhance mechanical properties, bioactivity, conductivity, and functional responsiveness [45]. However, in contrast to extrusion-based systems, the dominant constraints in vat photopolymerization are optical rather than rheological [46]. Nanoparticles can scatter or absorb incident light, altering photon transport within the resin and modifying photopolymerization kinetics. In highly filled systems, such as zirconium oxide loading of 50–70 wt.%, light penetration and cure depth are significantly reduced, while lateral light scattering broadens the polymerized area and compromises feature resolution [47]. These effects depend strongly on particle size, refractive index mismatch between the filler and the resin matrix, optical absorption characteristics, and dispersion state.

Beyond optical considerations, nanoparticle incorporation also affects the mechanical performance of the cured material. For instance, Maturi et al. developed graphene oxide modified with poly (butylene itaconate-co-adipate) (PBIA) as a photocurable system. This modification led to increases in elastic modulus and tensile strength (42% and 40%, respectively) [48]. However, increasing nanoparticle concentration typically raises resin viscosity, which can hinder resin flow, recoating efficiency, and overall process stability. Consequently, careful optimization of exposure parameters and resin formulation is required to maintain print fidelity and thermomechanical properties. Taking this into account, in photocurable nano-enabled systems, recent advances in design enable precise control over nanoarchitecture during fabrication. Macro chain transfer agents (macroCTAs) have been used for regulating internal morphology and structural topology [49]. Notably, mechanical properties can be tuned independently through macroCTA concentration, while enhanced swelling behavior enables the development of solvent-responsive systems. These findings highlight the potential of photocurable strategies for biomedical applications.

In two-photon polymerization, where highly focused lasers enable superior resolution, nanoparticle incorporation introduces further complexity due to nonlinear optical effects and enhanced scattering at high local energy densities. As a result, nanoparticle loading thresholds are often lower than in conventional SLA/DLP systems unless surface chemistry and optical properties are specifically engineered. In this study, Nuzhet et al. obtain total integration of high concentrations of nanoparticles up to 2 wt. %, above prior metal thresholds, by manipulating their surface and compatibility [50].

Despite these challenges, vat photopolymerization remains highly promising for fabricating mechanically reinforced and functional micro-scaffolds, provided that resin formulation and exposure parameters are carefully optimized [44].

The incorporation of nanoparticles into vat photopolymerization systems can significantly enhance mechanical, electrical, and functional properties. However, the addition also modifies the photopolymerization process directly by altering light transport. Specifically, nanoparticles introduce absorption and/or scattering phenomena that reduce cure depth, scattering length, and light penetration, imposing limitations on printing resolution. These effects are strongly dependent on particle type, concentration, and dispersion state [51,52].

Regarding cure depth, numerous studies have demonstrated that it can be reliably predicted through modified formulations of the Lambert-Beer law, in which nanoparticle concentration is incorporated as a critical parameter [53]. For instance, increasing the concentration of carbon nanotubes (CNTs) in the resin consistently leads to a reduction in cure depth. This trend is widely observed across different nanoparticle systems, including zirconia (ZrO_2_, 50–70 wt.%) [47], glass microspheres [54], and nanosilica (1–10 wt.%) [55], where higher nanoparticle loadings result in decreased cure depth. These phenomena are closely linked to changes in scattering length and light distribution. A higher refractive index of mismatch between nanoparticles and the surrounding matrix, as well as particle agglomeration, leads to a reduction in scattering length. This, in turn, increases lateral light spreading while decreasing through-thickness light penetration. Such behavior is directly influenced by particle size distribution and refractive index contrast. From a resolution standpoint, increased scattering generally deteriorates lateral resolution. Nevertheless, certain nanoparticles are intentionally introduced to reduce voxel size and enhance feature resolution, for example, by acting as radical quenchers [56,57,58].

#### 1.2.3. Powder-Bed Fusion: Selective Laser Sintering (SLS)

Selective laser sintering (SLS) is a powder-bed fusion technique in which a laser selectively fuses polymeric powders layer-by-layer. Unlike extrusion or vat-based methods, SLS does not require support structures, enabling the fabrication of complex porous architectures with high mechanical integrity. The solvent-free nature of the process can also be advantageous in terms of material purity and environmental issues.

For nanoparticle-reinforced bio-based polymers, however, several challenges arise. Achieving homogeneous nanoparticle distribution remains difficult, as differences in particle size, density, and surface energy may lead to segregation, resulting in inconsistent functional performance. For example, Fe_3_O_4_ nanoparticles have been successfully homogenized within porous poly(L-lactic acid)/thermoplastic polyurethane (PLLA/TPU) composites of moderate thickness, suggesting that processing parameters and scaffold geometry critically influence dispersion stability [59].

Furthermore, laser-material interactions are strongly influenced by nanoparticle optical absorption and thermal conductivity, which can alter sintering dynamics. High energy input during laser exposure may also induce thermal degradation of polymer matrices or structural changes in nanoparticles, potentially compromising the long-term performance of printed scaffolds. For instance, repeated reuse of Polyamide 12 powder has been associated with a decline in mechanical performance. Although dimensional accuracy is generally preserved, reductions in tensile strength, Young’s modulus, and elongation at break have been reported. These changes are typically accompanied by decreased bulk density and increased porosity, suggesting cumulative thermo-oxidative degradation [60]. However, in some cases, versatile polyethylene-derived biopolymers embedded with metal oxide nanoparticles and carbon black have been developed with high printability, tunable internal structure, and electrical conductivity [61].

Although SLS presents attractive possibilities for fabricating mechanically robust porous implants, its application to bio-based nanocomposites remains less explored due to these process limitations.

#### 1.2.4. In Situ Nanoparticle Formation During Printing

An emerging and innovative strategy in nanocomposite fabrication involves the in situ synthesis of nanoparticles during the 3D printing process. Rather than incorporating preformed nanoparticles into the printing material, precursor compounds are included in the polymer matrix or bioinks and converted into nanoparticles during or immediately after printing through thermal, photochemical, or chemical reactions [62,63].

This approach offers several advantages in terms of improved dispersion, reduced agglomeration, and enhanced interfacial compatibility. Moreover, it may enable spatial control of nanoparticle formation, facilitating the creation of functional gradients or localized active regions within a single construct. In fact, Bhattacharyya et al. obtained the modulation of amorphous calcium phosphate nanoparticles by adjusting the concentration of gelatin during the synthesis. This CNP GelMA nanocomposite is being understood as a bone tissue regenerative biopolymer [64].

Nevertheless, significant challenges remain. Controlling nanoparticle size, morphology, and distribution in situ can be complex, and precursor compounds may introduce cytotoxicity concerns. At early development stages for in vivo tests [65], preliminary in vitro validations are being conducted. In one such case, cytocompatibility was assessed using hFOB cells, demonstrating no detectable toxicity despite the incorporation of silver nanoparticles (AgNPs), which are commonly associated with dose-dependent cytotoxic effects. As a result, compatibility with living cells, bioactive molecules, and printing conditions must be rigorously evaluated [66]. At present, in situ nanoparticle formation remains largely at the proof-of-concept stage but represents a promising direction for future multifunctional biomedical constructs [67].

#### 1.2.5. Comparative Perspectives

Overall, the integration of nanoparticles into additive manufacturing processes introduces technology-specific constraints that must be considered from the earliest stages of material design. Extrusion-based systems are predominantly limited by rheological and thermal factors; vat photopolymerization techniques by optical interactions and curing kinetics; and powder-bed fusion processes by laser–particle energy coupling and powder homogeneity. Therefore, the choice of additive manufacturing technology should be aligned with the intended biomedical function, required resolution, permissible nanoparticle loading, and biocompatibility constraints. Rather than a secondary processing decision, printing technology constitutes a central design parameter in the development of high-performance nanocomposite bio-based materials.

## 2. Biopolymers for 3D-Printing: Mechanical, Rheological and Structural Properties

For hydrogel bioprinting systems (alginate, chitosan, hyaluronic acid, gelatin blends), the critical property centers on achieving appropriate yield stress (2–10 Pa for cell-laden systems, up to 300 Pa for acellular systems), shear-thinning behavior to enable flow through small nozzles, and rapid gelation post-deposition through temperature change or ionic cross-linking [68]. The storage modulus range of 200–1057 Pa provided sufficient structural integrity while maintaining cell viability [69,70]. These relatively low mechanical strength requirements (compressive moduli 2.7–186 kPa) reflect the intended applications in soft tissue engineering, where cell infiltration and remodeling are prioritized over immediate load-bearing capacity [71,72]. The challenge in this paradigm is not achieving high strength but rather maintaining dimensional accuracy with low-viscosity materials and ensuring long-term stability despite rapid degradation in physiological conditions.

### 2.1. Comparative Mechanical Properties

Hydrogel-based systems exhibited the lowest stiffness values. The pectin/genipin hydrogel showed an elastic modulus of 3–5 kPa under wet conditions at 37 °C in PBS, with no tensile or compressive data reported [73]. Similarly, nanocellulose/alginate/CaCO_3_ composites demonstrated tunable moduli from 1 to 30 MPa in the wet state, with a perpendicular modulus of 23.4 ± 1.1 MPa and tensile strengths between 4.2 and 6.1 MPa. Mid-range mechanical performance was observed in reinforced and protein-based systems [74]. Chitosan/cellulose nanofiber composites reached a Young’s modulus of 3.0 MPa, tensile strength of 1.5 MPa (stress at break), and strain at break of 75% [75]. Silk fibroin exhibited a modulus of 5.0 ± 0.1 MPa, tensile strength of 1.49 ± 0.14 MPa, and elongation at break of 104 ± 13% under hydrated conditions [76].

Table 1 summarizes the mechanical performance of different biopolymer systems used for 3D printing, highlighting Young’s modulus, tensile strength, strain at break, compressive properties, testing conditions, and references.

Chitosan in its neutralized state showed strong dependence on hydration and processing. Dynamic mechanical analysis (DMA) indicated a modulus of 2.3 MPa, while dried filaments reached 102 MPa. Tensile strength ranged from 95 MPa in the dry state to 6 MPa in the wet state, and strain at break reached 360% under wet conditions [77]. PLA/chitosan blends (10 wt.%) demonstrated modest mechanical modification relative to pure PLA, with stiffness increasing by 4–12% and tensile strength decreasing by 8–16%. Compressive behavior was reported as comparable to pure PLA under dry conditions [78]. The highest mechanical values were recorded for the acrylated epoxidized soybean oil (AESO), polyethylene glycol diacrylate (PEGDA), and nano-hydroxyapatite (AESO/PEGDA/nHA) composite exhibited a Young’s modulus of 6.6 GPa and tensile strength of 75.5 MPa in the dry state [79]. Similarly, polyhydroxybutyrate (PHB) showed a modulus of 3–3.5 GPa, tensile strength between 20 and 40 MPa, and strain at break of 5–10% in bulk form [80].

Compressive strength data are missing for most biopolymer systems, limiting comprehensive mechanical evaluation. This is critical because many target tissues primarily experience compressive loads in vivo. Standardized compressive testing under physiological conditions would improve functional assessment and comparability across studies.

### 2.2. Rheological and Printability Properties

Chitosan/cellulose nanofiber (CNF) formulations exhibit intermediate viscosities (100–500 Pa·s), enabling extrusion at relatively low pressures (0.15–0.77 bar) and comparatively high print speeds (40 mm/s). The use of nozzle diameters between 250 and 410 µm supports multilayer fabrication with stable filament formation. Notably, CNF reinforcement enhances structural integrity and shape retention, likely due to increased yield stress and improved filament self-supporting behavior after deposition [75]. Silk fibroin demonstrates substantially higher viscosity (≈1838 Pa·s), necessitating moderate extrusion pressure (0.4 MPa) and reduced deposition velocity (12 mm/s) to maintain continuous filament flow. A 200 µm nozzle enables relatively fine resolution, while cryogenic solidification immediately after extrusion provides rapid structural fixation, allowing up to four stacked layers with good dimensional stability. This system illustrates the role of thermal phase transition in compensating for high initial viscosity [76]. Other hydrogel systems based on pectin/genipin combination, although lacking reported viscosity values, operate at moderate print speeds (10 mm/s) and larger nozzle diameters (0.58 mm), achieving up to seven stacked layers. Quantitative printability metrics (uniformity factor U ≈ 1.02; printability index Pr ≈ 1.04) indicate near-ideal pore geometry and highly controlled filament spreading, suggesting a well-balanced viscoelastic response and effective post-deposition crosslinking [73].

Nanocellulose/alginate/CaCO_3_ composites are processed at 130–160 kPa and 25 mm/s using a 410 µm nozzle. These systems support fabrication of complex geometries with excellent shape fidelity, reflecting synergistic reinforcement by nanofibers and ionic crosslinking. The rheological profile likely includes pronounced shear-thinning and sufficient yield stress to prevent filament collapse, enabling free-standing architectures [74]. Neutralized chitosan (dry state) exhibits a broad processing window, tolerating extrusion pressures up to several MPa and print speeds ranging from very slow (0.1 mm/s) to high deposition rates (500 mm/s). Nozzle diameters between 30 and 500 µm allow both high-resolution printing and larger structural elements. The ability to achieve up to 30 layers indicates strong interlayer cohesion and post-processing stabilization, although dimensional changes may occur upon drying [77]. The AESO/PEGDA/nHA resin-based composite is extruded at approximately 592 kPa through a 0.6 mm internal diameter nozzle. Although detailed layer stacking data are not specified, printability is described as adequate, with some surface roughness observed. This behavior is consistent with photocurable systems in which structural stability is achieved primarily through post-extrusion UV crosslinking rather than purely rheological self-support [79]. Alginate/gelatin systems, extruded at 125–185 kPa and low print speeds (2 mm/s) using 400–600 µm nozzles, demonstrate structural integrity values between 87% and 99%. Despite the absence of reported viscosity data, these results indicate effective filament fusion and dimensional retention, likely mediated by ionic crosslinking and temperature-dependent gelation [81].

Overall, the table underscores that extrusion-based printability is governed by a tightly coupled interplay between rheological behavior (particularly viscosity and yield stress), extrusion pressure, deposition velocity, and nozzle diameter. Systems exhibiting optimized shear-thinning characteristics and rapid post-deposition stabilization consistently achieve superior multilayer stacking and geometric fidelity.

The following table (Table 2) provides a comparative overview of the rheological parameters, extrusion conditions, and printability performance of multiple biopolymer systems processed via extrusion-based 3D printing. It highlights how viscosity, extrusion pressure, deposition rate, and nozzle geometry collectively govern filament stability, layer stacking capability, and geometric fidelity.

### 2.3. Structural Properties

At the lowest level of network stabilization, physically gelled chitosan/cellulose systems demonstrate composition-dependent microstructural tunability. Increasing chitosan concentration reduces porosity, whereas CNF incorporation enlarges pore size and produces an interconnected fibrillar morphology. This dual effect reflects the balance between polymer chain density and fiber-induced structural organization. However, while multilayer constructs are achievable, the absence of long-term integrity assessment suggests that physical gelation alone may be insufficient for sustained mechanical stability under physiological conditions. The structural role of CNF here parallels reinforcement effects observed in other composite systems [75]. Table 3 summarizes the results.

Silk fibroin, although not chemically crosslinked, achieves structural consolidation through cryogenic self-assembly and β-sheet formation. The resulting macroporous, wrinkled morphology and enhanced fidelity under cryogenic printing indicate that conformational stabilization provides greater architectural robustness than simple physical gelation. Post-stretching further improves integrity, introducing anisotropic reinforcement similar in principle to fiber alignment in nanocellulose composites. Thus, both silk fibroin and nanocellulose-based systems exploit structural organization at the micro- to nanoscale to enhance macroscopic stability [76].

Covalent crosslinking, as exemplified by pectin/genipin, yields superior dimensional precision and post-processing stability. The tightly controlled pore size distribution (~100 µm range) and minimal geometric deviation demonstrate that chemical stability of the network minimizes relaxation and shrinkage. The presence of closed surface pores and open internal architecture suggests controlled diffusion gradients during crosslinking, contributing to structural stability. Compared to ionic systems, covalent crosslinking provides enhanced strength after lyophilization, highlighting the importance of permanent network formation for maintaining geometry during dehydration [73].

Ionic crosslinked systems, such as alginate-gelatin and nanocellulose/alginate/CaCO_3_, show high shape fidelity and structural integrity percentages approaching design values. However, their long-term performance diverges depending on reinforcement. Pure alginate-gelatin achieves high initial precision (87–99% integrity), yet lacks explicit functional load validation. In contrast, nanocellulose/alginate/CaCO_3_ withstands physiological pressure ranges (160–600 mmHg), demonstrating that particle dispersion and fiber alignment significantly enhance functional durability. This comparison underscores that ionic crosslinking alone ensures geometric retention, but reinforcement phases are necessary for mechanical stability [74,81]. Hybrid thermoplastic–hydrogel systems, particularly PCL/alginate-gelatin constructs, extend this reinforcement principle further. The thermally solidified PCL phase provides a mechanically stable framework that preserves overall porosity (~71–76%) while preventing degradation over 28 days. Here, structural integrity is decoupled from hydrogel stability and instead governed by the thermoplastic backbone, representing a transition toward mechanically dominant scaffolds [79].

Resin-based AESO/PEGDA/nHA systems represent the chemically most fixed architecture through UV photopolymerization. Functionalization enhances fidelity, and homogeneous nHA dispersion confirms effective microscale reinforcement [79]. However, the lack of reported porosity data limits comparison with hydrogel systems, suggesting that these composites prioritize mechanical robustness over highly porous, cell-instructive microarchitecture. Taken together, the results define a structural hierarchy governed by stabilization chemistry and reinforcement strategy. Physical gelation enables tunable porosity but limited validated durability; ionic crosslinking ensures high geometric precision but benefits substantially from fiber or particle reinforcement; covalent crosslinking and photopolymerization provide superior dimensional stability; and hybrid thermoplastic systems deliver the most sustained structural integrity. Nevertheless, inconsistent reporting of long-term degradation, load-bearing validation, and pore interconnectivity metrics prevents rigorous cross-system situations, highlighting a critical need for standardized structural characterization protocols in extrusion-based biopolymer research.

From a critical integrative perspective, the structural performance of these systems is tightly coupled to the crosslinking mechanism, reinforcement strategy, and phase architecture, starting from physically stabilized hydrogels to chemical and hybrid scaffolds.

## 3. Hybrids Nanocomposites for Additive Manufacturing

Hybrid NP-polymer compounds, commonly known as polymeric nanocomposites, are described as systems that combine an organic matrix with nanomaterials for improving their physical-chemical properties and applications [83,84,85]. This integration leads to synergistic improvements such as processability, lightness, and stability of polymers as well as the optical, catalytic, electronic, and magnetic properties of nanomaterials [86,87]. For achieving these improvements and the adequate integration of materials, it is key to control concentration, surface characteristics, and interfacial interactions of the nanomaterials and the biopolymeric matrix, among other aspects [88,89,90]. Specifically, reinforcement mechanisms are closely coupled with rheological changes. Papers on polymer nanocomposites and extrusion-based systems report that increasing nanoparticle concentration generally elevates zero-shear viscosity and enhances elastic response due to particle–particle and particle–matrix interactions [5,7]. While moderate increases in viscoelasticity can improve dimensional stability after deposition, excessive viscosity may reduce flow stability and narrow the processing window required for continuous extrusion [7].

Although the wide variety of properties and versatility shows potential for biomedical applications, these systems still face serious challenges such as nanoparticle aggregation, high cost of synthesis and functionalization, and concerns regarding toxicity, safety, and sustainability [83,87,91]. To assess the applicability of these materials in the biomedical field, some of their limitations are outlined below.

### 3.1. Agglomeration Phenomena and Interfacial Interactions

Achieving a homogeneous dispersion and distribution of nanoparticles/nanomaterials within a biopolymeric matrix is an essential requirement for successful extrusion-based 3D printing [92,93]. However, this constitutes one of the most challenging aspects in the production of polymeric nanocomposites, as nanoparticles of a wide variety of materials, such as metal, ceramic, and polymeric, present a strong tendency for agglomeration. This problem usually increases with nanoparticle concentration, which leads to a difficult compromise between the amount of the nanoparticle loaded and the possible loss of the characteristics of the nanoparticles [26,94,95]. Some common issues include the formation of clusters, sedimentation, and phase separation [95].

The mechanisms underlying these effects are of a diverse nature and may include incompatibility of intermolecular forces between nanomaterial and polymeric matrix, size-related effects, and surface change interactions. Regarding the nanoparticle, parameters such as size, morphology, and incorporation method followed for the synthesis of the nanocomposite play a decisive role in determining dispersion efficiency. An inadequate dispersion leads to problems during the printing process [92,94]. For example, one of the most frequent consequences is an increase in melt viscosity, which may require a higher extrusion pressure and, in the context of cell bioprinting, jeopardize cell viability. Fluctuations in viscosity are known to result in constructs with suboptimal structural quality. A particular critical issue is clogging, usually caused by agglomerates that obstruct the continuous flow of the material, interrupting the printing process, and directly affecting the geometry of the printed object [40,96,97].

Another of the main challenges lies in the poor compatibility between hydrophilic and hydrophobic surfaces, which are often approached by functionalization of the nanomaterial when possible. For instance, inorganic nanoparticles with a high hydrophilic nature would not be compatible with a hydrophobic polymeric matrix such as PLA [98,99]. In contrast, when interfacial adhesion is enhanced, this leads to significant improvements in mechanical strength and storage modulus (G’) and yield stress, parameters that are strongly correlated with filament stability and post-deposition shape retention [2,5], which is key for the behavior of the final nanocomposites [100,101,102].

The performance and properties of these hybrid systems depend largely on the quality of the interface established between the nanomaterial and the surrounding biopolymer matrix. Limitations associated with incompatible interfacial interactions are a common problem that usually results in lower mechanical integrity, reduced stability, aging behavior, and, overall, a decline in performance [88,103].

Polymeric nanoparticles are also generally prone to adhesion and degradation when exposed to high temperatures, sometimes required for their processing, though softer interfaces can be created by functionalization of the nanoparticle surface [94,104,105]. Some polymeric nanoparticles, such as nanocellulose, have been shown to be prone to clustering, settling, and irreversible aggregation during the drying process. This could be explained based on hydrophilic-hydrophobic incompatibility. Other polymers, such as PLA, PLGA, PMMA, and PLC, have been used for polymeric nanoparticle synthesis. These nanoparticles usually form agglomerates when combined with the polymeric matrix (chitosan, gelatin, and alginate), which leads to a heterogeneous dispersion and difficulties in printing. Functionalization, as well as different approaches for mixing, are commonly used to improve dispersion [105,106,107].

In the case of ceramic nanoparticles, known for their stiffness and bioactivity, clustering and phase separation are frequent, related to effects of nanoparticle size, electrostatic interactions, chemical incompatibility, and surface energy [94,108]. SiO_2_ nanoparticles are the most studied of ceramic nanoparticles. Apart from bioactivity, their size, shape, and porosity can be precisely controlled by the synthesis, which allows a nanoparticle design for a specific application [109,110]. Silica nanoparticles have been introduced in polymers such as PMMA or olefins, where their low surface compatibility led to the irreversible formation of agglomerates [111,112]. For instance, SiO_2_ nanoparticles incorporated in a polyimide matrix resulted in aggregation within the polymeric matrix, leading to loss of mechanical properties of the nanocomposite [113]. TiO_2_ nanoparticles are a type of ceramic nanoparticle that stands out due to their biocompatibility, stability, and photocatalytic activity. TiO_2_ nanotubes introduced in PS (polystyrene) led to aggregation problems due to interfacial compatibility between the surfaces of both materials and the high surface energy of the nanoparticles. In a study of TiO_2_ nanoparticles in PMMA, visible aggregates were observed after dispersion of the nanoparticles without salinization due to chemical incompatibility [114].

Regarding carbon-based materials, van der Waals forces and π-π stacking interactions are the main cause of agglomeration phenomena and formation of segregated networks [115,116]. Materials such as multi-wall carbon nanotubes and graphene nanoplatelets have been reported to present a strong tendency to aggregate formation associated with the interactions established. Moreover, the majority of pristine carbon nanostructures—carbon nanotubes and graphene—present high hydrophobicity and are very organophilic, while biopolymers are mostly. Naturally, this mismatch in surface energy and polarity results in poor interfacial adhesion and phase separation [115,116,117,118]. In this regard, carbon nanotubes, multiwalled carbon nanotubes in an aqueous matrix, have been reported to form aggregates and present very poor solubility, explained by the strong Van der Waals forces, π-π stacking phenomena, and high hydrophobicity. In addition, it is important to take into consideration the high aspect ratio of this type of nanomaterial, which further contributes to entanglement. These problems prevent the obtention of a uniform dispersion [119], leading to complex and heterogeneous networks. In addition, a study focused on the introduction of single-carbon nanotubes into a chitosan matrix describes poor dispersion of specific complexes due to strong intermolecular interactions with the biopolymer, leading to a weak binding and poor integration [120]. Carbon nanotubes have also been incorporated in starch, PVA, and PLCA, among others. In these cases, researchers have also reported agglomeration issues due to a combination of intermolecular interactions, hydrophilicity and hydrophobicity mismatch, high surface area, and concentration of carbon nanotubes [121,122]. Within carbon nanomaterials, graphene oxide, with abundant hydroxyl and carboxylic groups due to surface functionalization, is a strong alternative. Its modified surface presents higher hydrophilicity and higher compatibility with biopolymers, which leads to better dispersion and homogenous and stable solutions [123,124].

One of the primary advantages of nanodiamonds lies in their surface functionality. Their outer surface typically contains hydroxyl, carboxyl, or other oxygen-containing groups that facilitate hydrogen bonding and electrostatic interactions with polymer matrices [23]. In bio-based thermoplastics and hydrogel formulations, these interfacial interactions can enhance adhesion between filler and matrix, promoting more efficient stress transfer and potentially improving stiffness at relatively low filler contents. In extrusion-based additive manufacturing, such interfacial effects are particularly relevant because filament stability and interlayer cohesion are strongly dependent on viscoelastic behavior and polymer chain mobility.

Despite their promising characteristics, nanodiamonds also present practical challenges. Strong particle–particle interactions promote aggregation, which reduces effective surface area and limits interfacial reinforcement efficiency [23]. Achieving stable dispersion often requires surface modification or optimized mixing strategies, potentially increasing processing complexity and cost. Furthermore, at elevated filler fractions, restriction of polymer chain mobility may contribute to increased brittleness in thermoplastic matrices, affecting toughness and long-term mechanical reliability.

Metallic nanoparticles typically suffer from clustering or non-homogeneous dispersions, mainly explained by their high density, different surface properties, electrostatic incompatibilities, and cohesive forces. Factors such as concentration, pH, and ionic strength-induced assembly must be considered [93,125]. Silver nanoparticles have been introduced in polymers such as gelatin, alginate, or silk fibroin, where they have undergone different degrees of aggregation, from moderate to medium, or concentration and temperature dependent [126,127]. For instance, silver nanoparticles in various polymeric matrices suspended in PBS have shown medium-dependent aggregation related to the ionic strength of the PBS solution. Gold nanoparticles have also been combined with polymers such as chitosan or cotton fibers for biomedical applications [128,129]. For example, gold nanospheres have been incorporated in alginate for bioprinting, which led to issues due to agglomeration of the nanoparticles due to the presence of Ca^2+^ in the polymer and its high ionic strength [130]. In addition, gold nanoparticles in chitosan and organic solvent resulted in severe aggregation, leading to the loss of their optical and photothermal properties [131]. In a similar way, magnetic nanoparticles have been shown to aggregate in polymeric media such as DMSA or PMA-alt-ODE [8,132]. Lastly, ZnO nanoparticles in PLA have been shown to agglomerate due to poor interfacial compatibility [133,134].

Overall, the efficiency reinforcement of hybrid nanosystems utilized in extrusion-based additive manufacturing is strongly determined by specific interfacial mechanisms and nanoparticle type. In the case of polymeric nanoparticles and those of biological origin, dominant interactions with the matrix are based on hydrogen bonds and chain intertwining, which notably affects the material viscosity and printing stability. Nonetheless, at high concentrations, these mechanisms are prone to favor aggregation [95,135,136]. Ceramic nanoparticles are known to reinforce the matrix by the contrast between rigidity and surface energy interactions, though their low chemical compatibility and electrostatic effects tend to cause the formation of aggregates and phase separation in the absence of surface functionalization [137,138]. In the case of carbon-based nanomaterials, π-π stacking and van der Waals interactions allow the generation of percolated networks and the improvement of electric and mechanical properties, yet they also cause strong agglomeration and dispersion issues, especially in biopolymeric matrices [139,140]. Metallic nanoparticles can interact with the matrix through electrostatic forces and ionic force-related mechanisms, which leads to their dispersion being very sensitive to the chemical environment [141]. In this context, identification and control of dominant surface mechanisms become essential to achieve balance among mechanical reinforcement, processability during impression, and long-term performance in the manufacturing of hybrid nanosystems.

### 3.2. Effects of Nanoparticle Loading on Rheology and Printability

The incorporation of nanomaterials into biopolymer inks fundamentally alters their rheological properties, which may be beneficial or a disadvantage when working with 3D printing. While nanoparticles are often added to enhance mechanical properties and improve shape fidelity, their inclusion usually involves significant printability constraints [142,143]. A fundamental issue is the increase in viscosity produced by the introduction of the nanomaterials in the polymeric matrix, which leads to the use of higher extrusion pressures and becomes more problematic at higher concentrations. This is an important challenge for bioprinting applications, as generally, a high shear stress negatively affects cell viability [142].

Nozzle clogging is one of the most frequently mentioned printability limitations that occur as a consequence of nanoparticle incorporation. The formation of clusters and agglomerates can easily obstruct the orifices of the printer nozzles, which leads to a failed extrusion. These rheological modifications result in changes in filament cohesion, uncontrolled fiber spreading, and loss of resolution, which ultimately influence the integrity of the printed object [94,144,145,146].

As each nanoparticle type presents different characteristics, they may lead to specific rheological limitations. For instance, while ceramic nanoparticles are known to significantly increase stiffness and viscosity and generate excellent shear-thinning properties, particularly in bone-oriented constructs, higher concentrations of nanoparticles require higher shear stress that may affect cell viability as well. In addition, poor layer adhesion has also been reported [147,148]. Some studies report that modified nanoclays and nanoHA (hydroxyapatite) particles may occasionally cause clogging or poor dispersion [149,150,151].

Regarding polymeric nanoparticles, although they also induce an increase in viscosity, yield stress, and stiffness, they can cause swelling and may present some incompatibility with the polymeric matrix. Some related printability issues are the need for a higher extrusion pressure, formation of aggregates, and a lower resolution due to the high surface tension, often requiring a defined and limited interval of concentrations for adequate printability [92,106]. For instance, polyaniline nanoparticles introduced in photopolymer matrices have led to high yield stress and poor flow at low shear. The consequences are an increase in viscosity concentration-dependent, with very low thresholds, as well as irregular layer deposition [152].

Metallic nanoparticles have a characteristic high density and thermal conductivity, which can affect melt flow behavior. In fact, insufficient temperature during the extrusion procedure can become an issue and lead to agglomeration that reduces the mechanical properties of the obtained filaments [93,153,154]. For instance, gold nanoparticles incorporated in polymeric matrices for optical fiber probes report a concentration-dependent aggregation tendency, limiting the number of nanoparticles that can be introduced in the material, which also limits the magnitude of their optical and thermal properties [155].

Lastly, carbon-based nanomaterials lead to a strong increase in viscosity as well as elastic modulus, sometimes inducing solid-like behavior [156,157]. When well dispersed, CNTs and graphene-based fillers can enhance tensile strength and elastic modulus through improved stress transfer at the polymer–nanofiller interface [18,19]. Reviews on graphene–polymer composites highlight that even low loadings can deliver measurable mechanical reinforcement, although the magnitude of improvement varies with functionalization, matrix chemistry, and dispersion route [18]. In hydrogel-based extrusion systems, GO is particularly relevant because its surface functional groups promote interactions (e.g., hydrogen bonding and electrostatic interactions) with polymer chains, which can increase storage modulus (G′), enhance yield stress, and improve filament stability and shape fidelity after deposition [2,158]. This rheological reinforcement can be beneficial for printability, provided that the formulation remains within an operational shear-thinning window [2,158].

Nevertheless, the incorporation of carbon-based nanomaterials also introduces characteristic limitations. Graphene derivatives and CNTs tend to agglomerate due to strong interparticle interactions, and maintaining homogeneous dispersion remains a major challenge in polymer matrices [18]. Agglomeration reduces effective interfacial area, limits stress transfer efficiency, and may introduce defects that act as stress concentrators, thereby compromising toughness and mechanical reliability [16,18].

In addition, network formation associated with percolation commonly leads to sharp increases in zero-shear viscosity and elastic modulus, reflecting the development of a particle network within the matrix [5,6]. In extrusion-based processing, such abrupt rheological transitions can narrow the printable window, increase pressure requirements, and reduce flow stability, particularly as filler content approaches or exceeds the percolation threshold [5]. Studies have documented that high concentrations of multi-walled carbon nanotubes in PLA result in a very high viscosity and, therefore, increased resistance to flow, making printing impossible [144,159].

From a rheological perspective, nanodiamonds may significantly influence flow behavior during extrusion. Due to their high surface energy and strong interparticle interactions, they can increase zero-shear viscosity and storage modulus (G′), especially in hydrogel systems where secondary interactions contribute to network formation [22,23]. Moderate increases in viscoelasticity may enhance filament shape fidelity and reduce collapse after deposition. However, excessive loading or inadequate dispersion can narrow the printable window, increase extrusion pressure requirements, and introduce flow instabilities, similar to other high-surface-area nanofillers.

The dispersion quality of nanoparticles can be evaluated by microstructural factors such as the size distribution of aggregates, the area or volume fraction occupied by the aggregates, or the dispersion index obtained by image analysis (SEM/TEM) and dispersion techniques (DLS/SAXS), combined with stability parameters against sedimentation. These indicators show a direct correlation between the effective reinforcement fraction and the available interfacial surface area for stress transfer [95,160]. In general terms, a more homogeneous dispersion increases the effective surface area and the contact polymer reinforcement, which translates into a higher storage modulus and an increase in yield stress for a given load, along with a larger elastic contribution in oscillation assays. On the contrary, poor dispersion, associated with the presence of big agglomerates, reduces the accessible surface area and the efficiency of stress transfer, in addition to introducing effects that act as stress concentrators, which may decrease the material toughness and limit the mechanical reinforcement expected from the nominal filler fraction [95,161,162]. From a rheological standpoint, dispersion quality is reflected in variations in the xero-shear viscosity, in the onset of pseudoplastic behavior, and in the appearance or intensity of a low-frequency storage modulus plateau associated with the formation of particle networks. These effects tend to depend more on the effective percolated fraction than on the nominal nanoparticle content, which explains why formulations with identical loadings may exhibit significantly different rheological and mechanical behavior when their degree of dispersion is not equivalent [163,164].

The distinctions summarized in Table 4 emphasize that nanoparticle selection cannot be guided by isolated performance metrics. Instead, material suitability emerges from the interplay between interfacial interactions, rheological behavior, and process stability. These coupled effects become particularly critical when addressing formulation design and extrusion parameter optimization, as discussed in the following sections.

### 3.3. Surface Functionalization Strategies to Improve Compatibility

Surface modification of nanoparticles is a fundamental strategy to reduce the many limitations that arise from their incorporation into biopolymeric matrices, such as low dispersity and low compatibility at the interface. To improve their rheology and printability, their properties have been modified using chemical approaches [94,121,165]. One of the most widely used is the carboxylation of the nanomaterial surface, which leads to a higher dispersion due to electrostatic repulsion. In addition, silanization is also commonly used in silane-based materials as it helps establish a bridge between the inorganic and organic parts of the composite. PEGylation is an additional approach that improves the hydrophilicity and biocompatibility of the system [159,166].

Regarding carbon-based nanomaterials, surface chemistry modification is key for improving biocompatibility and dispersion. It is usual to apply covalent functionalization involving the attachment of chemical groups such as carboxyl, hydroxyl, or amine to the nanomaterial surface, improving its hydrophilicity and compatibility with the polymer. It is important to keep in mind that this functionalization may partially disrupt the sp^2^ structure of the material, which may lead to a decrease in the mechanical and electrical properties [167,168,169]. An alternative is the use of non-covalent functionalization, which is less destructive. This includes applying surfactants (CTAB and SDS, etc.) or biopolymers that are wrapped around the nanomaterials [170,171,172]. Use of surfactants is usually performed in very low amounts due to their cytotoxicity, which is problematic for biomedical applications. Some promising biopolymers are chitosan, gelatin, silk fibroin, and nanocellulose, especially as “green” dispersants, stabilizing carbon nanomaterials through electric repulsion, steric hindrance, or more specific interactions (cation-π, for instance). For example, carbon nanotubes and multi-walled carbon nanotubes previously functionalized by means of carboxylation and oxidation have been dispersed in PLA and chitosan, resulting in improved compatibility and more stable dispersions [173,174]. As an example of the non-covalent approach, SDS, CTAB, and Pluronic have been used to stabilize single-walled carbon nanotubes and multi-walled carbon nanotubes in alginate, starch, and chitosan, leading to better dispersion and stability [169,175,176]. In addition, biopolymers such as chitosan, gelatin, and nanocellulose have been wrapped around carbon nanotubes and graphene as a strategy to improve dispersibility and better preserve the carbon nanotubes’ properties [177,178].

In a similar way, functionalization agents for silanization are typically applied to ceramic and metallic nanomaterials to improve their compatibility. An interesting example is the functionalization of titanium oxide with phenyl and thiol groups to facilitate hydrophilicity and dispersion, leading to an important increase in the Young modulus of the material. When working with ceramic and metallic nanoparticles that are introduced into organic resins, silanization is commonly applied. During this process, silane coupling agents help build a molecular bridge between the inorganic nanoparticle surface and the organic polymeric matrix. This approach leads to a stronger bonding, facilitating the dispersion, reducing aggregate formation, and improving biocompatibility [179,180,181]. SiO_2_ nanoparticles that have undergone silanization and have been introduced in cellulose acetate or starch-chitosan blends have shown good compatibility, reduced interfacial voids, and a far more homogeneous dispersion. In addition, some more complex nanoparticles with a metallic nucleus and silica coating may also benefit from this surface modification strategy to improve dispersion and compatibility with the polymeric matrix [182,183].

Another method usually applied to a wide range of nanomaterials is polymer coating or grafting, such as PEGylation. It consists of adding polymer chains on the surface of the nanomaterial to enhance hydrophilicity and to provide steric hindrance, resulting in less agglomeration. This method not only provides an improved dispersion and colloidal stability, but it is also applied to reduce non-specific protein adsorption and to improve biocompatibility [184,185]. This strategy has been widely applied to a wide variety of nanoparticles, such as gold nanoparticles [186,187], silver nanoparticles [188], silica nanoparticles [189,190], or titanium nanoparticles [191], to increase dispersibility, achieve a more homogeneous combination, and reduce agglomeration and interface incompatibility. To give examples of other polymers, TiO_2_ nanoparticles have been grafted with PMMA and carbon nanotubes with PS for lower aggregation [192,193].

Nevertheless, functionalization and modification approaches for surface modification present their limitations. Often, chemical treatments may result in a more complex process, which leads to a higher cost and an additional challenge of scale-up. Moreover, the chemical alteration of the nanomaterial surface may cause different interactions with biomolecules, where the possible consequences must be adequately assessed. Biocompatibility constitutes an additional design constraint in biomedical applications. Graphene-based materials show a strong dependence of biological response on lateral size, oxidation state, surface chemistry/functionalization, and impurity content [20]. While functionalization can improve dispersion and interfacial compatibility within bio-based matrices, excessive modification may reduce electrical conductivity by disrupting the conjugated structure, which can undermine the intended multifunctional advantage [18].

**Figure 1:**
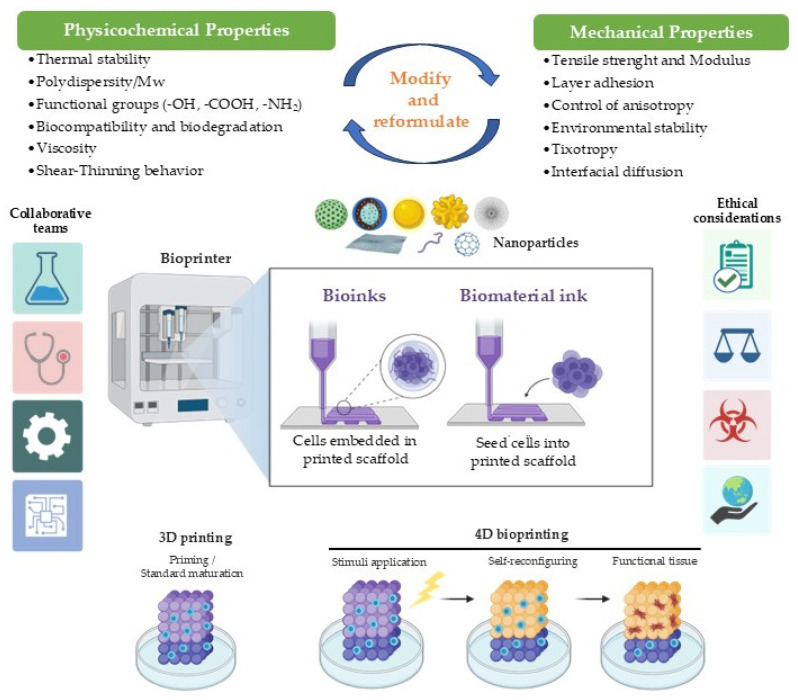
Conceptual framework of bioinks and biomaterial ink for 3D and 4D printing. Schematic overview of the physicochemical properties and mechanical parameters, including viscosity, shear-thinning behavior, interfacial diffusion, and anisotropic mechanical response, emphasizing interdisciplinary collaboration and ethical considerations.

Ultimately, it is important to consider the general lack of systematic studies in the scientific literature regarding this topic, which makes it difficult to determine the real benefit of each functionalization strategy. Figure 1 presents the physicochemical properties and mechanical parameters, including viscosity, shear-thinning behavior, interfacial diffusion, and anisotropic mechanical response, emphasizing interdisciplinary collaboration and ethical considerations.

## 4. Biomedical Applications

Among the emerging fabrication methods, 3D printing holds significant promise for advanced biomedical applications such as the production of biosensors, bioimaging, drug delivery devices, and tissue engineering strategies, among others. Its ability to create customized structures offers high potential for improving performance and functionality. Moreover, the versatility of 3D–5D printing also supports the use of diverse substrate types, thereby broadening potential applications. This section reviews key biomedical applications of nanocomposite-based 3D printing constructs.

Advances in biomedical applications have driven a conceptual shift in additive manufacturing, marking the evolution from three-dimensional (3D) to four- and five-dimensional (4D, 5D) printing. The new concept of 4D printing introduces the dimension of time, as Tibbitts of the Self-Assembly Lab at the Massachusetts Institute of Technology (MIT) described in 2013 [194]. This technology prints constructs to undergo controlled, programmed transformations in response to external stimuli (e.g., temperature, moisture, pH, light, magnetic fields, or biochemical signals). The two major approaches used in 4D bioprinting include shape morphing and functional transformation. Other approaches that have been proposed include biomimicry, self-assembly, and in vivo 4D bioprinting [195]. 4D bioprinting typically consists of three principal steps: (1) pre-4D bioprinting design, including the 4D bioprinting path design and smart bioink preparation/development; (2) 4D biofabrication of the designed constructs by using a proper bioprinter; and (3) post-4D bioprinting processing, including stimuli-induced shape change and maturation of the tissues and organs. In 5D printing, five degrees of freedom are achieved through the coordinated movement of the printer’s print head and the object platform, allowing the fabrication of complex curved layers instead of traditional flat layers. This approach enables the production of parts with enhanced structural strength and geometrical complexity without requiring additional material, which is particularly suitable for biomedical applications [196].

The incorporation of nanoparticles into bio-based polymer matrices can act as active fillers, transducers, or functional modifiers. Depending on their composition and morphology, nanoparticles are capable of changing shape, mechanical properties, or functionality when exposed to specific triggers. For example, magnetic nanoparticles can enable remote actuation under external magnetic fields, while photothermal nanoparticles allow localized heating and shape transformation upon light irradiation. Similarly, bioactive or degradable nanoparticles can modulate time-dependent changes in mechanical properties or porosity thanks to matrix remodeling.

### 4.1. Biosensors

The growing demand for the detection, monitoring, and quantification of specific chemical species and reactions has driven significant advances in sensor development [197]. In the field of biosensing, nanomaterials derived from biomaterials have attracted considerable attention due to their ability to enhance specificity, sensitivity, and cost-effectiveness [198]. Typically, a biopolymer-based biosensor comprises a bioreceptor and an electrode or transducer, where signal detection occurs at the bioreceptor, and the binding of an analyte generates a measurable increase in the electrochemical signal at the output, as observed in electrochemical biosensors [199]. Their ability to detect low analyte concentrations, combined with portability, rapid response, and low cost, has made them valuable tools in diverse fields such as healthcare, environmental monitoring, and food safety [200].

Several conductive polymers, including polyindole, polyfuran, polyaniline, and polycarbazole, are commonly employed in biosensing applications in combination with biocompatible flexible substrates and adhesive biopolymers such as silicone, hydrogels, and natural biomaterials (e.g., silk fibroin and cellulose). Additionally, the incorporation of nanomaterials such as carbon nanotubes, graphene, and metallic nanoparticles into inks with precisely tuned rheological properties has enabled the fabrication of high-performance electrodes with improved conductivity and enhanced sensing capabilities [200,201].

For instance, the research group led by Professor Janegitz has explored 3D-printed biosensors for SARS-CoV-2 detection; notably, Silva et al. [202] developed a graphene–PLA-based genosensor incorporating gold nanoparticles for virus detection via cDNA hybridization monitored with a redox probe.

A 3D-printed electrode cassette fabricated by fused deposition modeling for detecting dengue and chikungunya was developed by Sharma et al. The incorporation of silver nanoparticles to paper-based electrodes enabled rapid and sensitive electrochemical detection of dengue and chikungunya antigens over a linear range of 1 × 10^2^ to 1 × 10^6^ ng/mL, delivering results in 20 s with stability up to 30 days and successful identification of coinfections in blood serum samples [203].

### 4.2. Tissue Engineering

Tissue regeneration represents one of the most extensively investigated applications of nanocomposite-based 3D/4D/5D printing with unprecedented precision and control that closely mimic native tissues and organs. Advanced 4D and 5D printing technologies can adapt scaffold architecture in response to cellular activity or changes in the surrounding tissue, enhancing integration and tissue maturation. They also allow dynamic adjustment of structural strength and geometrical complexity, enabling the fabrication of patient-specific drug delivery devices [196]. These approaches also facilitate the creation of complex architectures capable of encapsulating drugs, growth factors, or therapeutic nanoparticles, thereby ensuring precise spatial and temporal control over drug release [204].

Bio-based polymers commonly used in 3D printing often lack sufficient mechanical strength or bioactivity when used alone, limiting their effectiveness in regenerative applications. As a sophisticated solution, the incorporation of nanoparticles such as hydroxyapatite, bioactive glass, calcium phosphates, or carbon-based nanomaterials has been shown to enhance the mechanical properties and biological performance of printed scaffolds. At the nanoscale, these fillers can mimic aspects of the native extracellular matrix, providing biochemical patterns that influence cellular behavior. Additive manufacturing enables the design of scaffolds with controlled porosity, interconnected pore networks, and spatial gradients in composition. This architectural control is particularly valuable for replicating the heterogeneity of native tissues and for guiding tissue ingrowth and vascularization. However, the effectiveness of these systems is strongly dependent on nanoparticle dispersion and bonding within the polymer matrix, underscoring the importance of material-process compatibility [205].

Currently, most studies focus on the design and development of scaffolds aimed at promoting the regeneration of bone, cartilage, and soft tissues. A major challenge in this field lies in meeting the specific functional requirements of these tissues, necessitating scaffolds that not only provide adequate mechanical support but also actively enhance cell adhesion, proliferation, and differentiation [206].

In bone tissue engineering, for example, nanoparticle-reinforced scaffolds have demonstrated improved osteoconductivity and mineralization compared to unfilled polymeric counterparts. Additionally, recent studies have demonstrated that ZnO-integrated 3D-printed hydrogels significantly improve the mechanical properties of the bone constructs, accelerating tissue regeneration and promoting bone and vascular reconstruction in defect areas [207]. In this context, a novel strategy for the treatment of critical-size bone defects was developed using a high-temperature fused deposition modeling 3D-printed scaffold composed of poly(lactic-co-glycolic acid) (PLGA) reinforced with silicon nitride (Si_3_N_4_) nanoparticles and loaded with bone marrow-derived mesenchymal stem cell (BMSC) microgels encapsulated in GelMA hydrogel [208]. The incorporation of Si_3_N_4_ improved the scaffold’s hydrophilicity, mechanical properties, and bioactivity, while the BMSC microgels enhanced cell survival and therapeutic efficacy. In vitro and in vivo studies demonstrated that the composite scaffold promoted osteogenesis, angiogenesis, and anti-inflammatory responses, leading to enhanced vascularization and bone regeneration in rat femoral defect models within eight weeks, highlighting its potential for bone tissue engineering applications.

Additionally, the combination of 3D/4D printing biopolymers with nanomaterials based on silver, gold, and/or zinc has recently gotten much attention in wound dressing applications due to their inherent antimicrobial properties [209]. Thus, printed objects containing PLA-AgNPs have exhibited significant antibacterial behavior against *Staphylococcus aureus*, *Escherichia coli*, and *Pseudomonas aeruginosa* without chemical changes to the matrix polymer [210]. On the other hand, the antiviral activity is likely attributed to the presence of polyanionic sulfate groups, whose strong negative charge facilitates interactions with positively charged molecules on the surfaces of receptor cells [211].

### 4.3. Drug Delivery: Controlled vs. Targeted

The integration of advanced bioprinting techniques with nanocomposite materials has created new opportunities for the development of precise and customizable drug delivery systems with enhanced therapeutic efficacy. The distinctive properties of nanocomposites, including improved drug-loading capacity, tunable release profiles, and superior mechanical strength, further expand their biomedical potential due to the additional properties provided by nanomaterials in polymers, such as their electrical, thermal, and magnetic characteristics [212]. Additionally, combining biopolymers with such nanomaterials generates multifunctional hybrid systems that enhance site-specific delivery, optimize administration routes, prolong therapeutic activity, and enable precise spatiotemporal control over drug release [213]. In addition, functionalized smart nanoparticles incorporated into 3D-printed matrices can also respond to external stimuli such as pH, temperature, magnetic fields, or light, triggering a precise temporal- and spatial-drug release.

While controlled and targeted delivery offers significant therapeutic advantages, it also introduces some important challenges, such as additional complexity in terms of material design, printing reproducibility, and safety evaluation. In both cases, the value of combining nanoparticle multifunctionality with the control of pharmaceutical dose fabrication by 3D printing is showcased. In this context, the selection of appropriate nanoparticles is crucial, as they may ensure uniform dispersion and distribution within the matrix and optimize processing parameters to achieve compatibility and efficient incorporation.

In this context, the dosage form and administration route must also be taken into consideration to evaluate the efficacy of the system. For instance, Beck et al. developed 3D-printed tablets by incorporating polymeric nanocapsules loaded with deflazacort into devices fabricated via fused deposition modeling using poly(ε-caprolactone) (PCL) and Eudragit^®^ RL100 filaments, with or without mannitol as a channeling agent [214]. Partially hollow tablets (50% infill) achieved higher drug loading (0.27% *w*/*w*) and faster release, halving the drug release half-life from 20 to 10 min. This approach demonstrates an innovative method for transforming nanocapsule suspensions into solid dosage forms, offering a versatile 3D printing strategy for personalized drug delivery systems.

Recently, a multifunctional 3D-printed scaffold based on polycaprolactone/nano-hydroxyapatite (PCL/HA) was developed and coated with zein to enable sustained release of tetracycline hydrochloride for bone regeneration in infection-prone environments [215]. The scaffold exhibited improved mechanical performance and interconnected porosity suitable for trabecular bone applications, while the zein coating reduced burst release and allowed controlled drug delivery for up to 14 days. The system showed effective antibacterial activity against *Staphylococcus aureus* and *Escherichia coli* and supported osteoblast-like cell adhesion and viability, demonstrating its potential as a cost-effective and clinically relevant platform for bone tissue engineering.

As an example of biomedical applications in cancer treatment, Mirdamadian et al. fabricated Eudragit^®^ L100-55 filaments containing alginate NPs encapsulating oxaliplatin (OP-NPs) by the hot-melt extrusion method [216]. The active ingredient was formulated as NPs to improve oxaliplatin antitumor activity, tumor targetability, and safety profile. These OP-NP-loaded filaments were then utilized to produce 3D-printed tablets using a fused deposition modeling printer. The antitumor effects of the 3D-printed tablets containing OP-NPs were found to be remarkable and comparable to those of intravenous OP solutions, while demonstrating a superior safety profile. On the other hand, Cho et al. demonstrated the precise fabrication of 3D-printed nanogel disks containing paclitaxel and rapamycin, which prevented premature gelation during storage and avoided an initial burst release in dissolution studies [217]. These nanogel disks enabled effective intraperitoneal drug delivery in ES-2-luc ovarian cancer xenograft mice, showing therapeutic efficacy and preventing postsurgical peritoneal adhesions.

### 4.4. Bioimaging

Bioimaging represents a modern advancement in the medical field, employing digital technologies to visualize biological processes in real time and to characterize the three-dimensional structure of specimens [218]. This non-invasive approach enables detailed analysis using techniques such as light and electron microscopy, fluorescence imaging, ultrasound, X-rays, magnetic resonance, and positron emission. In this context, polymer nanocomposites have shown considerable promise, providing enhanced contrast and incorporating functional nanoparticles, such as quantum dots and gold nanostructures, into 3D-printed constructs for real-time tumor visualization. These multifunctional systems support the integration of therapeutic and diagnostic functions, advancing the development of theragnostic platforms, while key attributes for high-performance bioimaging include low cytotoxicity, strong photostability, efficient luminescence, cost-effectiveness, bioconjugation potential, and good water dispersibility. Moreover, the scalability of 3D printing makes these systems suitable for precision medicine; however, challenges remain in optimizing material properties, ensuring biocompatibility, and achieving efficient large-scale production [197].

**Figure 2:**
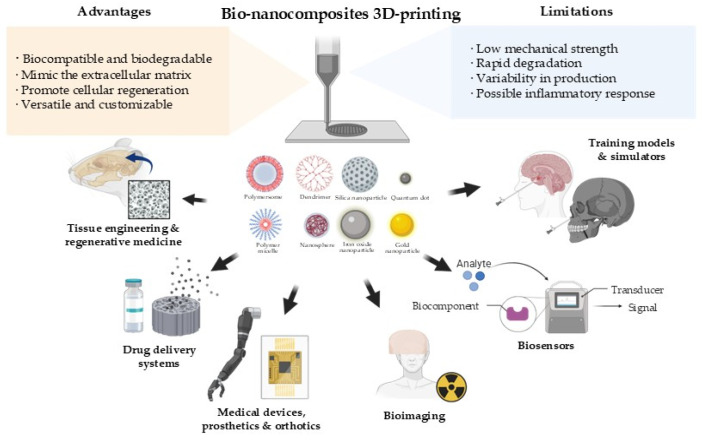
Applications, advantages, and limitations of biopolymers in 3D printing. Overview of the principal advantages and current limitations of biopolymers in 3D printing, alongside representative biomedical applications.

Conventional 3D-printed implants require fabrication followed by surgical implantation, a complex procedure associated with tissue damage, infection risk, and prolonged recovery. Noninvasive 3D printing offers a promising alternative by enabling the in situ fabrication of structures directly within affected areas or onto exposed tissue surfaces, reducing the need for invasive surgical interventions [219]. The recent study developed by Ana Iglesias-Mejuto et al. [220] is an example of an in situ bioimaging 3D-nanocomposite printing scaffold. In this work, infrared-responsive 3D biomaterials were formulated by integrating highly fluorescent upconversion nanoparticles into dual-porous, 3D-printed alginate aerogels. The resulting porous scaffolds were capable of supporting tissue regeneration and enabling real-time implant monitoring, as in vivo studies over three weeks confirmed. Figure 2 shows an overview of the principal advantages and current limitations of biopolymers in 3D printing, alongside representative biomedical applications.

## 5. Challenges and Limitations in the Clinical Translation of 3D-Printed Biopolymers

Despite the remarkable progress achieved in nanoparticle-integrated additive manufacturing, a substantial gap persists between laboratory-scale demonstrations and routine clinical implementation. While preclinical studies frequently report enhanced mechanical performance, bioactivity, and therapeutic functionality, translation into medical practice remains constrained by intertwined material, manufacturing, regulatory, and environmental challenges. These limitations extend beyond isolated technical issues and reflect the intrinsic complexity of combining nanotechnology with additive manufacturing in biologically regulated environments.

### 5.1. Compatibility, Processability and Reproducibility

The primary technical bottleneck in nanoparticle-integrated additive manufacturing lies in achieving a robust balance between nanomaterial incorporation and processability. While the biological interaction of 3D-printed biopolymers represents a double-edged sword, where the incorporation of nanoparticles offers therapeutic benefits, it simultaneously profoundly modifies the physicochemical behavior of polymer matrices. These modifications extend beyond simple compositional changes and directly influence rheological properties, thermal stability, crystallization behavior, optical response, and interfacial dynamics at multiple length scales.

Consequently, the enhancement of functional performance is frequently accompanied by a narrowing of the processing window required for reliable and reproducible printing. In extrusion-based techniques such as FDM, agglomeration at higher volume fractions can act as a stress concentrator, weakening the composite rather than reinforcing it. Increased melt viscosity and altered shear-thinning behavior can further compromise filament flow stability, dimensional accuracy, and interlayer adhesion [221]. Similarly, as shown in vat photopolymerization processes such as SLA, high nanoparticle loading may increase light scattering and resin viscosity, disrupting photopolymerization kinetics, reducing cure depth, and impairing spatial resolution [222,223]. In powder-based platforms, disparities in particle size, density, and surface energy may promote segregation phenomena and uneven laser-material interactions, ultimately affecting consolidation and microstructural homogeneity.

A fundamental trade-off therefore emerges between maximizing nanoparticle loading to achieve functional gains and preserving dimensional accuracy, structural fidelity, and surface quality. Achieving homogeneous dispersion is critical, yet technically challenging, particularly as nanoparticle concentration increases [97]. Alterations in flow behavior, curing dynamics, and powder homogeneity may compromise printability in a platform-dependent manner, underscoring the need for formulation strategies tailored to each manufacturing route.

Although nanoparticles are generally incorporated to enhance mechanical strength, stiffness, or bioactivity, reinforcement is not guaranteed. Poor dispersion, weak interfacial adhesion, or excessive loading may introduce stress concentration sites, restrict polymer chain mobility, and ultimately reduce mechanical performance. These effects become particularly critical in load-bearing applications, where reliability and fatigue resistance are essential.

Furthermore, additive manufacturing inherently generates anisotropic microstructures due to layer-by-layer deposition [97]. Heterogeneous nanoparticle distribution may exacerbate directional mechanical variability, complicating predictive modeling of implant behavior under physiological loading conditions.

In addition, processing conditions may induce physicochemical changes in nanoparticles, potentially altering their intended functionality. Thermal exposure during extrusion, photochemical reactions during resin curing, and high-energy laser irradiation in powder-based systems can lead to surface oxidation, chemical degradation of functional coatings, or morphological transformations such as incomplete fusion and formation of agglomerates. These microstructural irregularities may compromise mechanical integrity, accelerate degradation, or create unpredictable release profiles in drug-release systems.

For nanoparticles designed to deliver drugs, modulate immune responses, or provide imaging contrast, maintaining structural and chemical stability throughout fabrication is essential to ensure predictable therapeutic performance after implantation [224]. Ensuring homogeneous dispersion and minimizing process-induced defects, therefore, requires robust formulation design, optimized parameter control, and advanced in situ monitoring strategies.

Beyond technical compatibility, economic and logistical factors further constrain translation. As noted in the recent literature, high-quality nanomaterials with controlled size distribution and surface functionalization remain costly and may exhibit batch-to-batch variability. Even subtle differences in particle morphology or surface chemistry can significantly influence composite performance and degradation behavior. In decentralized or hospital-based printing models, ensuring consistent quality traceability becomes even more demanding.

Scaling from laboratory-scale prototyping to industrial manufacturing intensifies these challenges [225,226,227]. It will require not only material innovation but also strategies that guarantee dimensional stability and sufficient mechanical robustness during mass production. Maintaining dimensional tolerances, mechanical reproducibility, and dispersion stability across large production volumes requires standardized protocols, validated quality control frameworks, and compliance with Good Manufacturing Practice (GMP) guidelines [228].

These guidelines present logistical and financial challenges. Specifically, this compliance represents a major hurdle for customized or patient-specific implants. Technologies such as FDM and SLA must maintain tight precision. Thus, even minor deviations in layer adhesion or post-processing deformation can compromise implant fit or drug release kinetics. For that reason, advances in low-temperature curing routes and solvent/thermal free-processes, coupled with adhesion pre-treatments and barrier interlayers, demonstrated and established scalable production. In addition, sterility assurance and traceability are more difficult to standardize. Furthermore, postmarket surveillance and long-term biocompatibility data are limited, making it difficult to study the chronic effects of residual monomer, degradation byproducts, or nanoparticle release in vivo.

### 5.2. Biological Safety and Long-Term Performances

The biological translation of 3D-printed biopolymers represents one of the most critical and complex barriers to clinical implementation [229]. While nanoparticles confer enhanced bioactivity, antimicrobial functionality, imaging capability, or controlled drug delivery, their interaction with biological systems introduces uncertainties that extend beyond conventional polymeric biomaterials.

Nanoparticles possess unique physicochemical properties such as high surface area, surface reactivity, and nanoscale dimensions that can influence cellular behavior in ways not observed for bulk materials. Although numerous in vitro studies report acceptable cytocompatibility [230], concerns remain regarding dose-dependent cytotoxicity, oxidative stress induction, DNA damage and genotoxicity, and/or inflammatory and immune responses.

Particular attention is required for metal and metal-oxide nanoparticles (e.g., Ag, Au, ZnO, and TiO_2_), which are widely investigated for antimicrobial or theranostic applications. While these materials demonstrate clear therapeutic benefits, their long-term biological fate and potential accumulation in sensitive tissues remain insufficiently characterized. For instance, ZnO nanoparticles have been shown to enhance antibacterial activity in 3D-printed chitosan–agarose scaffolds [231]. However, their ion release kinetics and cytotoxicity profiles must be profoundly studied [232].

In resin-based systems, metal-oxide reinforcement nanoparticles have been used. While some of them have demonstrated antimicrobial functionality and cytocompatibility [43,233], the presence of residual photoinitiators and unreacted monomers represents additional sources of cytotoxicity, asking for optimization of curing protocols and safer initiator chemistries [234]. Even when bulk mechanical performance is satisfactory, trace chemical residues may compromise biological safety.

In this sense, a persistent translational gap exists between in vitro biocompatibility testing and in vivo performance [229]. Standard cytotoxicity assays provide short-term viability data but often fail to capture chronic exposure effects, nanoparticle release dynamics, and protein corona formation [65], immune system modulation, and organ-level biodistribution. In fact, Haixin Jiao et al. printed hydrogels with sawdust biomass waste [235]. As a result, the process minimizes endotoxin and (1,3)-β-D-glucan, promoting higher cell viability. For nanocomposites designed for cancer therapy, neurological applications, or immunomodulation, these limitations are particularly relevant. Constructs incorporating liposomes, dendrimers, magnetic nanoparticles, or metallic nanocarriers may exhibit promising therapeutic functionality in controlled environments, yet long-term biocompatibility and degradation behavior in complex physiological systems remain inadequately understood. In fact, in vitro studies have been performed, obtaining no significant toxicity, suggesting promising biocompatibility [236]. But even then, some biopolymers, emphasizing metal or metal oxide NPs (Ag, Au, ZnO, and TiO_2_), require careful toxicity and degradation products assessment.

The degradation profile of nanocomposite biomaterials is more complex than that of conventional polymers. As the polymer matrix undergoes hydrolytic or enzymatic degradation, embedded nanoparticles may be gradually released into surrounding tissues. Nanoparticle liberation may occur through surface erosion, bulk degradation, matrix swelling, or mechanical fragmentation, depending on the polymer architecture and physiological conditions. Variability in nanoparticle dispersion within the matrix further complicates the prediction of degradation kinetics and mechanical stability.

For load-bearing or long-term implants, uncontrolled release or structural weakening could compromise both safety and functionality. For example, biodegradable polymers such as poly(lactic-co-glycolic acid) (PLGA) undergo hydrolysis of ester bonds under physiological conditions. These by-products, even if regarded as biocompatible, may lead to systemic distribution and accumulation in organs such as the liver, spleen, or lungs during long periods of time [237].

Long-term biopolymers introduce additional uncertainty regarding mechanical durability, structural integrity, and sustained therapeutic activity. The potential accumulation in specific organs must be considered. Recent in vivo studies have demonstrated that enzymatic hydrolysis of polylactic acid can generate nanoplastic oligomers in murine models, triggering localized inflammatory responses with metalloproteinases [238]. Also, PLGA nanoparticles can induce endothelial dysfunction [239].

In emerging 4D-printed systems, where constructs are designed to undergo time-dependent transformations in response to physiological stimuli, safety evaluation becomes even more demanding. Small variations in nanoparticle loading or dispersion may significantly alter actuation behavior or degradation dynamics. Ensuring reliable long-term performance requires rigorous validation across extended timeframes.

### 5.3. Regulatory Adaptation to Complex and Dynamic Models

Beyond material optimization and biological validation, the clinical deployment of 3D-printed nanocomposite biomaterials is strongly conditioned by regulatory adaptation, manufacturing governance, and environmental responsibility. These dimensions are often underestimated during early-stage research but become decisive barriers during translation.

Agencies such as the U.S. Food and Drug Administration (FDA) and the European Medicines Agency (EMA) require extensive documentation of material composition, degradation profiles, biocompatibility, and reproducibility, but current regulatory frameworks were primarily developed for conventional medical devices characterized by static composition and well-defined performance profiles [222]. In contrast, 3D-printed nanocomposites are compositionally heterogeneous, process-sensitive, and, in some cases, dynamically responsive. Regulatory approval requires comprehensive documentation of nanoparticle physicochemical characterization, batch reproducibility and dispersion homogeneity, degradation kinetics and by-product analysis, long-term biocompatibility, and manufacturing traceability [240]. Variability in nanoparticle size distribution, surface chemistry, or dispersion state may directly affect mechanical behavior and biological safety, impacting patient safety. Consequently, even minor formulation changes may necessitate renewed validation, increasing regulatory complexity. Furthermore, as novel bioinks emerge with immunomodulatory functions or enhanced immune surveillance in cancer immunotherapy, regulators must update frameworks to assess long-term host interactions, particularly for immunocompromised patients [224].

This challenge becomes more pronounced in the context of patient-specific manufacturing and decentralized printing models, where ensuring reproducibility, sterility, and traceability is inherently more demanding than in centralized industrial production.

The emergence of stimuli-responsive and 4D-printed nanocomposites introduces additional regulatory uncertainty. Traditional device evaluation is largely based on static mechanical and biological properties. However, constructs designed to evolve over time—through shape morphing, controlled release, or immunomodulatory activation—require assessment across their entire functional lifecycle.

### 5.4. Environmental Impact of 3D-Printed Biopolymer

The increasing incorporation of nanomaterials into bio-based polymers for biomedical 3D printing raises important questions regarding their environmental impact across the material life cycle. While the primary focus of nanoparticle-reinforced scaffolds is clinical performance, potential environmental exposure may occur during material production, processing, degradation, and disposal. In this context, these materials represent a relevant case study due to their growing use and their unique physicochemical properties.

The interaction of nanomaterials with the environment involves complex and dynamic processes that may result in both beneficial and adverse effects. In soil, nanoparticles demonstrate a notable capacity to interact with heavy metals such as chromium through mechanisms including surface complexation, electrostatic interactions, redox reactions, and biologically mediated transformations. This ability not only facilitates the removal of toxic substances but also contributes to their transformation into less harmful forms or stabilization as colloids.

However, the behavior of these materials is generally not benign. Upon release into the environment, these materials can persist and tend to accumulate over time, undergoing aggregation, reduction, or partial decomposition, processes that strongly influence their mobility, bioavailability, and reactivity. For instance, certain graphene-based materials, such as graphene nanosheets, have been reported to enhance the accumulation of arsenite in soil, increasing its availability and therefore toxicity. Similarly, graphene oxide is known to exhibit significant persistence in air and water, raising concerns regarding long-term exposure and accumulation. Considering aquatic ecosystems, graphene oxide and similar nanomaterials with pollutants such as zinc and cadmium show changes in the metabolic and physiological processes of aquatic organisms. In fish, these interactions can lead to changes in metabolic rates and biological responses, highlighting the potential ecological risk associated with them.

From the perspective of biomedical additive manufacturing, these findings emphasize the importance of considering environmental safety alongside biocompatibility. During the 3D printing process, volatile organic compounds (VOCs) and other nanoparticles can be released [241]. These air pollutants can induce toxicity effects on the respiratory (asthma or allergic rhinitis), cardiovascular, and nervous systems from occupational exposure [242,243]. Specifically, residual photoinitiators and unreacted monomers used for SLA/DLP-based nanocomposites raise concerns regarding cytotoxicity and regulatory approval.

The degradation of bio-based polymers and the possible release of embedded nanomaterials after clinical use remain insufficiently understood. So, it is essential to ensure that the biomedical and environmental benefits of bio-based nanomaterials are not offset by unintended ecological consequences. In a recent study, a photopolymer resin was described [244]. By modifying internal bonds in lipoates, the material can be recycled, reused, and reprinted in a circular way. As a result, this lipoate derivative becomes a biodegradable material offering safety advantages.

### 5.5. Translational Perspective of 3D-Printed Biopolymer

From a translational perspective, several critical challenges, such as scalability, cost, and reproducibility, continue to limit the clinical and preclinical biomedical applications of 3D bio-based polymers. At the manufacturing level, current bioprinting platforms still face important constraints in achievable printing speed, spatial resolution, and, notably, the ability to maintain consistent and reproducible material flow during nozzle stop–start events—limitations that are often amplified by the incorporation of nanoparticles [245]. The integration of nanomaterials into 3D manufacturing printing also introduces a set of interconnected challenges that must be carefully addressed to enable safe, reliable, and clinically relevant applications. A major concern relates to the potential toxicity of certain nanomaterials, which may restrict their suitability for pharmaceutical and biomedical use depending on their composition, size, surface chemistry, and degradation behavior. In addition, achieving a homogeneous dispersion of nanoparticles within printable formulations remains technically demanding, as particle agglomeration can result in nozzle clogging, structural defects, and batch-to-batch variability in the final printed dosage forms. The inherently high surface area and reactivity of nanomaterials further raise safety considerations, including increased health risks during material handling and processing, particularly with respect to inhalation or unintended exposure. From a manufacturing standpoint, scaling up nanomaterial production while preserving consistent quality and physicochemical properties continues to be problematic, as material behavior may change when moving from laboratory-scale synthesis to large-scale manufacturing. Furthermore, precise control over printing parameters is essential to minimize residual stresses and microstructural heterogeneities that could compromise the mechanical integrity, performance, and dose accuracy of printed pharmaceutical products [246].

In parallel, the restricted availability of printable, FDA-compliant biopolymers, together with regulatory uncertainty surrounding nanocomposite bioinks, represents a major barrier to translation and commercialization. These issues are further compounded in clinical applications of 3D bioprinting, particularly in the development and commercialization of bioprinted tissue equivalents, where additional challenges include ensuring the long-term longevity and functionality of printed tissues, meeting tissue-specific biomechanical and physiological requirements, and addressing often underestimated ethical and legal considerations related to patient safety, data ownership, and regulatory responsibility [97].

These limitations are especially evident in emerging applications such as 3D-printed in vitro cancer models, where biological variability, lack of standardized protocols, and difficulties in maintaining long-term cultures that recapitulate tumor progression restrict their robustness as preclinical tools, further complicated by the intrinsic heterogeneity across cancer types [247].

**Figure 3:**
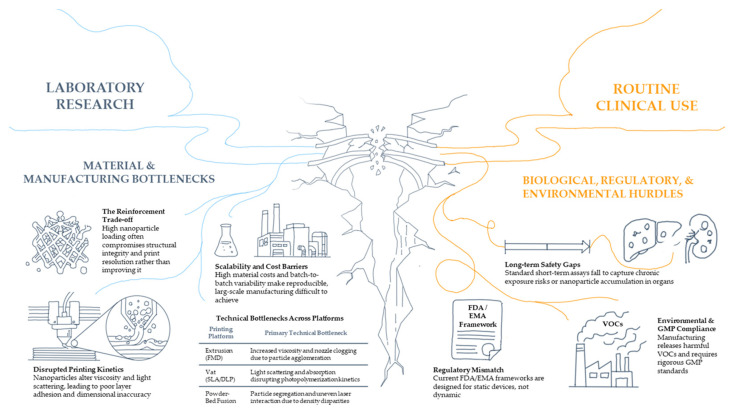
Translational bottlenecks from laboratory research to routine clinical use. Schematic representation of the material, manufacturing, biological, regulatory, and environmental challenges limiting the clinical translation of biopolymers.

Looking ahead, future directions in 3D bioprinting are expected to encompass transformative approaches, including bioprinting under microgravity conditions to overcome gravitational and diffusion-related constraints, as well as the integration of artificial intelligence for bioink optimization, process control, and predictive modeling of tissue maturation [248]. Overcoming these challenges will require coordinated advances in nanomaterial design, formulation engineering, and printer process control, alongside the implementation of robust safety guidelines and standardized manufacturing workflows that support regulatory compliance and industrial scalability. The following figure, Figure 3, represents the translational bottlenecks from laboratory research to routine clinical use.

## 6. Future Perspectives

### 6.1. Green Nanomaterials

The future development of green nanomaterials for additive manufacturing will depend less on the demonstration of isolated proof-of-concept syntheses and more on the systematic integration into standardized, scalable, and regulated biopolymers. While the adaptation of the green chemistry principles formulated by Paul Anastas and John Warner has conceptually reshaped nanoparticle synthesis—prevention, atom economy, less hazardous chemical synthesis, designing safer chemicals, safer solvents and auxiliaries, design for energy efficiency, use of renewable feedstock, reduced derivatives, catalysis, design for degradation, real-time analysis for pollution prevention, and inherently safer chemistry for accident prevention, their translation into reproducible 3D-printable systems remains at an early stage.

Green pathways have been developed for a wide range of nanomaterials relevant to printable systems, including metallic, metal oxide, chalcogenide nanoparticles, carbon dots, and lignin-based nanostructures [249,250,251]. These approaches typically involve coupled processes of bioreduction, nucleation, growth, and stabilization occurring simultaneously in aqueous and low-energy environments. By typically utilizing aqueous media, avoiding toxic organic solvents, and employing natural, non-hazardous reducing and capping agents, these methods drastically reduce the generation of toxic waste and by-products, thereby minimizing pollution and overall environmental impact [249,252]. While mechanistically complex, such processes yield nanoparticles with functionalized surfaces, facilitating their incorporation into inks and nanocomposite formulations.

In the context of 3D printing, green nanomaterials are not standard entities but functional fillers or active components embedded within polymeric, hydrogel, or resin-based matrices. The integration of green nanomaterials into printable formulations further requires a formulation-driven perspective. Rather than optimizing isolated nanoparticles, future approaches should be tuned to synthesis parameters according to rheological targets, crosslinking chemistry, curing mechanisms, and printing modality. In this sense, biomolecule-assisted synthesis may offer particular advantages due to its improved compositional definition and compatibility with hydrogel-based matrices [250]. Biomolecules such as chitosan and ascorbic acid function as eco-friendly reducing and capping agents while simultaneously serving as ink components or matrix modifiers, enabling improved control over nanoparticle dispersion, interfacial interactions, and crosslinking behavior, supporting the development of ink-ready biopolymers [253].

A critical next step for the field is the transition from qualitative toward quantifiable sustainability metrics. Future research should incorporate life cycle assessment (LCA), energy consumption analysis, and waste stream quantification to evaluate whether biologically mediated routes offer measurable environmental advantages over optimized chemical synthesis. Without such metrics, the risk of superficial green labeling remains significant.

Plant extracts have obtained particular attention due to their rich phytochemical composition, including polyphenols, terpenoids, and alkaloids, which can act as multifunctional reducing and capping agents [254]. In printable formulations, these chemicals may contribute to enhanced colloidal stability and reduced nanoparticle aggregation within polymer matrices. Moreover, the avoidance of harsh reagents, such as hazardous reducing agents like sodium borohydride or hydrazine and aggressive stabilizers like CTAB, mitigates risks tied to chemical handling, storage, and disposal. However, equally important is the need for standardization and quality control frameworks. Plant-mediated and microorganism-assisted synthesis inherently introduces compositional variability that challenges batch reproducibility [249,252]. Future efforts should prioritize the definition of critical quality attributes (e.g., nanoparticle size distribution and surface ligand density…) and the establishment of purification protocols compatible with European requirements. For example, endotoxins, pyrogens, or other immunogenic cellular components, if microbial cultures are used, might harbor allergens or other bioactive compounds that could be undesirable or even detrimental in the final biopolymer application, especially if intended for biomedical use.

Another emerging direction involves the rational engineering of the bio-derived surface corona. Nanoparticles synthesized using biological extracts are inherently enveloped by a layer of biomolecules from the source [255]. The unique bio-capping layers present on green-synthesized gold (AuNPs), selenium (SeNPs), and iron oxide nanoparticles can significantly facilitate drug loading, for instance, with anticancer agents like doxorubicin, and subsequently improve their bioavailability and targeted delivery to pathological sites [251]. In tissue engineering, green biopolymers are increasingly incorporated into scaffolds to actively promote cell adhesion, proliferation, and differentiation, or to impart crucial antimicrobial properties, supporting functional tissue regeneration [256]. While naturally occurring, capping layers can enhance colloidal stability and biocompatibility; they may also introduce immunogenic or regulatory uncertainties. Future work should focus on controlled biofunctionalization strategies that preserve sustainability benefits while ensuring chemical definition and regulatory traceability.

From a translational standpoint, regulatory requirements will play a decisive role. Green origin does not inherently mean biological safety, and systematic toxicological evaluation will be necessary for clinical adoption [249]. Collaborative efforts between materials scientists, toxicologists, and regulatory experts will therefore be essential to bridge laboratory innovation and biomedical deployment.

Ultimately, the field must evolve from demonstrating environmentally benign nanoparticle synthesis toward the construction of fully integrated, standardized, and performance-validated green bio-polymer platforms. Achieving this shift will require interdisciplinary design frameworks that simultaneously address sustainability, printability, scalability, and biological performance. Only through such convergence can green nanomaterials move from conceptual promise to practical implementation in advanced 3D-printed biomedical systems.

### 6.2. Artificial Intelligence in Nano-Enabled Additive Manufacturing

#### 6.2.1. Data-Driven Formulation Design and Predictive Modeling

To overcome the trade-offs between resolution, speed, and material properties, the integration of Artificial Intelligence (AI) into the development of nanoparticle-reinforced bio-based materials for 3D printing has emerged as a critical enabler. AI and machine learning algorithms are increasingly recognized as a key strategy to address the intrinsic complexity of these systems. Unlike conventional polymeric formulations, nanocomposite bioinks involve a high-dimensional design space in which not only material composition but also nanoparticle characteristics, processing parameters, and biological performance are strongly interdependent. In this context, data-driven approaches offer a rational alternative to empirical trial-and-error methodologies, which are often inefficient, poorly reproducible, difficult to scale, and cost-dependent.

Machine learning (ML) techniques are particularly well suited to capture nonlinear relationships between formulation variables and printing outcomes, enabling predictive modeling, optimization processes, and accelerated material discovery [257,258]. Instead of manually exploring combinations of polymers, nanoparticles, and functional additives, ML models can screen large compositional spaces to identify robust formulation strategies with desirable combinations of mechanical performance and biological response. In this study, by integrating multilayer perceptron (MLP) architectures with complementary modeling strategies, the HydroThermo-MLP framework demonstrated strong predictive capability for rheological responses across varying shear and thermal conditions in HAMA/GelMA systems [259]. This approach illustrates how data-driven approaches can reduce empirical iterations in hydrogel optimization. Also, Chen et al. employed several algorithms, such as Decision Trees, Random Forest, and Deep Learning, to analyze 210 ink formulations based on 16 biomaterials, achieving high predictive accuracy and precision, positioning machine learning as a powerful tool to accelerate the development of this sector [260]. Rather than replacing experimental work, these early-stage tools complement it by guiding experimental design, reducing the number of iterations required, and improving consistency across printing platforms.

A major challenge in extrusion-based printing of nanocomposite bio-inks lies in balancing viscosity, yield stress, structural recovery, and cytocompatibility. The incorporation of nanoparticles frequently alters rheological behavior in ways that classical constitutive models fail to describe, particularly in systems containing anisotropic or two-dimensional nanomaterials. Data-driven models are therefore especially valuable in correlating rheological descriptors with print fidelity and scaffold performance. Also, well-trained ML models have demonstrated strong potential for predicting simultaneously printability and rheological response, correlating geometric descriptors with mechanical and biological performance. In one study, Wang et al. employed backpropagation neural networks and random forest algorithms to predict key hydrogel properties relevant to printability. Their models accurately captured viscosity variations across different shear rates and polymer rates [261].

In biomedical applications, the performance of 3D-printed constructs depends not only on material composition but also on scaffold architecture. Parameters such as pore size, porosity gradients, anisotropy, and lattice topology critically influence mechanical behavior, nutrient diffusion, cell migration, and tissue integration. In this context, Chen et al. developed a Bayesian optimization framework to determine optimal processing parameters such as layer height, nozzle travel speed, and dispersing pressure in direct ink writing (DIW) for the fabrication of presurgical organ models with intricate geometries [262]. By that, the model enables simultaneous improvement of geometric fidelity, dimensional accuracy, and porosity control.

Such approaches illustrate how AI-driven optimization can support the rational tuning of process parameters to achieve targeted architectural and functional outcomes, thereby facilitating the development of advanced and application-specific scaffolds.

#### 6.2.2. AI-Assisted Process Monitoring and Quality Control

While predictive modeling supports formulation design, maintaining structural fidelity during printing remains a critical challenge. Nanocomposite bio-inks are often highly sensitive to environmental fluctuation, leading to defects such as nozzle clogging, layer delamination, or geometric distortion. These issues become particularly relevant in biomedical applications, where reproducibility and regulatory traceability are essential [258,263,264].

AI-driven monitoring systems, including computer vision, integrated with high-resolution imaging techniques (e.g., CT scanning), enable real-time detection of printing defects and deviations from intended geometry [263,265]. By correlating process parameters with structural outcomes, these systems can facilitate corrective interventions during fabrication, enhancing consistency and reducing material waste. Such quality control frameworks are especially valuable for nanoparticle-reinforced bio-based systems, where small variations in dispersion or rheology may translate into significant structural heterogeneity [266]. For example, Kathiravan et al. applied AI-driven techniques, including convolutional neural networks (CNNs) and reinforcement learning (RL) algorithms, to address key challenges in additive manufacturing. Their results suggest that deep-learning-based vision systems enable adaptive process optimization, reducing the total average material waste from 17% to 8%. Their system achieved a defect detection accuracy of 98.6% in fused deposition modeling (FDM), with false positive and false negative rates remaining below 5% [267]. These findings illustrate the potential of AI-assisted monitoring to enhance manufacturing efficiency while improving reproducibility and minimizing resource consumption.

Beyond defect detection, AI-assisted monitoring contributes to improving traceability and data documentation, both of which are critical for regulatory approval of medical devices and implantable constructs. In this sense, AI enhances not only technical performance but also the reliability and scalability of nano-enabled additive manufacturing platforms.

#### 6.2.3. Digital Twins in Nano-Enabled Additive Manufacturing

The digital twin paradigm extends this capability toward fully integrated, adaptive manufacturing. A digital twin can be defined as a dynamic virtual representation of a physical system that integrates real-time process data, physics-based models, and data-driven algorithms to simulate, predict, and optimize system behavior. Digital twins (DTs) can be interpreted as the convergence of AI-driven predictive modeling and real-time monitoring strategies. Unlike machine learning models, which typically operate offline, digital twins enable continuous bidirectional data exchange between the physical printing systems and their virtual counterparts [268,269]. Thus, a digital twin is not merely a predictive model but a fully closed-loop control framework that integrates prediction through AI-based systems, sensor-driven monitoring, and adaptive control, enabling real-time adjustment.

In the context of nano-enabled bio-inks and printable nanocomposites, digital twins offer the potential to bridge formulation design, process parameters, and final construct performance within a unified computational environment [270,271]. Such capability is particularly relevant to address the intrinsic variability of nanocomposite bioinks. For instance, fluctuation in viscosity due to nanoparticle dispersion or temperature variations could be dynamically compensated through real-time adjustment of extrusion pressure or deposition speed [270].

Similarly, digital twins could facilitate the simultaneous optimization of process parameters and scaffold architecture by linking in situ sensor data with predictive models of mechanical and biological performance. This is particularly relevant in biomedical applications, where scaffold properties such as porosity, anisotropy, and microstructural organization directly influence cell behavior and tissue integration [272,273]. For instance, digital twin strategies are being explored in the fabrication of organoids and complex tissue constructs, where not only the structural geometry but also the functional performance must be replicated. In this context, DTs can integrate biological, mechanical, and process-related data to support the generation of physiologically relevant three-dimensional structures. Digital twins may thus contribute to reducing experimental trial and error, minimizing material waste, and improving scalability. Thus, key considerations in sustainable advanced manufacturing.

Although still at an early stage in bio-fabrication research, the integration of digital twin strategies with AI-driven materials discovery and process optimization represents a promising direction toward more robust, adaptive, and sustainable nano-enabled additive manufacturing platforms. Their implementation in biofabrication and bioprinting remains at an early conceptual stage or in early proof-of-concept stages. Moreover, recent efforts are focused on the development of modular and reusable DT architectures that can be adapted across different additive manufacturing platforms, facilitating scalability and broader adoption [274].

#### 6.2.4. Limitations in AI-Assisted Nanocomposites

Despite the promise, AI and ML approaches face several important limitations that must be critically acknowledged [275]. First, model performance is highly dependent on the quality, size, and standardization of training datasets. In the field of nanocomposite-based 3D printing, available datasets are often fragmented, non-standardized, and generated under heterogeneous experimental conditions [264]. Second, many ML models function as “black boxes”, providing limited insight into the underlying physical or biological mechanism. This lack of understanding generates low acceptance in clinical and regulatory contexts. Third, models trained for specific material or techniques may not readily transfer to other systems, necessitating careful validation. Adaptive systems, while technically attractive, may require new regulatory frameworks to ensure patient safety and long-term reliability.

Future progress will likely emerge from hybrid strategies that integrate machine learning with physics-based modeling and rigorous experimental validation. The development of explainable models may improve trust, interpretability, and regulatory acceptance. In addition, the establishment of a standardized database for nanocomposite formulations will be essential to unlock the full potential of data-driven design.

The evolution toward 4D printing further amplifies the importance of computational intelligence. Predicting time-dependent transformations in nanoparticle-reinforced systems involves complex interactions between material composition, architecture, and environmental stimuli. AI-driven approaches, particularly when embedded within digital twin architectures, may enable the rational design of constructs that are not only patient-specific in geometry but also dynamically adaptive in function. Such integration aligns with the broader vision of personalized medicine with individual biomedical devices, bridging material science, additive manufacturing, and digital medicine.

## Figures and Tables

**Table 1:** Comparative mechanical performance of biopolymer-based materials used in 3D printing. Summary of Young’s modulus, tensile strength, strain at break, compressive properties (when available), and testing state for selected biopolymer systems. “N. r.” indicates not reported.

Biopolymer System	Young’s Modulus/Elastic Modulus	Tensile Strength	Strain at Break	Compressive Strength/Modulus	Testing State	Ref.
Chitosan/Cellulose	3.0 MPa	1.5 MPa (stress at break)	75%	N. r.	N. r.	[75]
Silk fibroin	5.0 ± 0.1 MPa	1.49 ± 0.14 MPa	104 ± 13%	N. r.	Hydrated	[76]
Pectin/Genipin	3–5 kPa	N. r.	N. r.	N. r.	Wet, 37 °C, PBS	[73]
Nanocellulose/Alginate/ CaCO_3_	1–30 MPa (wet); 23.4 ± 1.1 MPa perpendicular	4.2–6.1 MPa	N. r.	N. r.	Wet	[74]
Chitosan (neutralized, dry)	2.3 MPa (DMA); 102 MPa (dried filaments)	95 MPa (dry); 6 MPa (wet)	360% (wet)	N. r.	Dry and wet states	[77]
PLA/Chitosan (10 wt.%)	Stiffness increased 4–12% vs. pure PLA	Decreased 8–16% vs. pure PLA	N. r.	Comparable to pure PLA	Dry	[78]
AESO/PEGDA/nHA	6.6 GPa	75.5 MPa	N. r.	N. r.	Dry	[79]
PHB	3–3.5 GPa	20–40 MPa	5–10%	N. r.	Bulk	[80]

**Table 2:** Rheological parameters, extrusion conditions, and printability performance of representative biopolymer systems processed by extrusion-based 3D printing. Comparative overview of viscosity, extrusion pressure, printing speed, nozzle diameter, layer stacking capability, and shape fidelity for selected biopolymer inks processed via extrusion-based 3D printing. “N. r.” indicates not reported.

Biopolymer System	Viscosity	Extrusion Pressure	Printing Speed	Nozzle Diameter	Layers Achieved	Shape Fidelity Assessment	Ref.
Chitosan/Cellulose	100–500 Pa·s	0.15–0.77 bat	40 mm/s	250–410 µm	Multilayer	Good, improved with CNF	[75]
Silk fibroin	1838 Pa·s	0.4 MPa	12 mm/s	200 µm	4 layers	Good cryogenic solidification	[76]
Pectin/Genipin	N. r.	N. r.	10 mm/s	0.58 mm	Up to 7 layers	U = 1.02, Pr = 1.04	[73]
Nanocellulose/ Alginate/ CaCO_3_	N. r.	130–160 kPa	25 mm/s	410 µm	Complex structures	Excellent high fidelity	[74]
Chitosan (neutralized, dry)	N. r.	0–4.1 MPa	0.1–500 mm/s	30–500 µm	Up to 30 layers	Good complex shapes achieved	[77]
AESO/PEGDA/nHA	N. r.	592 kPa	N. r.	0.6 mm ID	N. r.	Adequate despite surface roughness	[79]
Alginate/gelatin	N. r.	125–185 kPa	2 mm/s	400–600 µm	N. r.	Structural integrity 87–99%	[81]

**Table 3:** Crosslinking mechanisms and structural performance of 3D-printed biopolymer scaffolds. Overview of crosslinking strategies and associated structural characteristics, including porosity, morphology, shape fidelity, and structural integrity of representative biopolymer scaffolds. “N. r.” indicates not reported.

Biopolymer System	Crosslinking Mechanism	Porosity/Pore Size	Shape Fidelity	Surface Morphology	Structural Integrity	Ref.
Chitosan/Cellulose	Physical gelation	Porosity decreases with CHI concentration; pore size increases with CNF	Good multilayer constructs	Fine network with interconnected fibrils	Not assessed long-term	[75]
Silk fibroin	Cryogenic self-assembly	Macropore structure	Enhanced by cryogenic printing	Wrinkled	Enhanced by post-stretching	[76]
Pectin/Genipin	Genipin crosslinking	108 µm surface; 104 µm cross-section	6.7% height deviation, 1.9% diameter deviation	Closed surface pores; open internal pores	High-dimensional stability post-lyophilization	[73]
Nanocellulose/ Alginate/ CaCO_3_	CaCO_3_/Gdl, CaCl_2_, or HCl	N. r.	High	Uniform CaCO_3_ distribution. Fiber alignment	Withstands 160–600 mmHg pressure	[74]
PLA/Chitosan (10 wt.%)	None	500 µm pore size	Adequate	Rough surface due to chitosan particles	Slow degradation	[78]
AESO/PEGDA/nHA	UV photopolymerization	N. r.	Improved with functionalization	Good dispersion of nHA by SEM	N. r.	[79]
Alginate-gelatin	CaCl_2_ ionic crosslinking	Porosity similar to design for 500–600 µm nozzles	High precision	N. r.	87–99% integrity	[81]
PCL/alginate-gelatin	CaCl_2_ (hydrogel); thermal (PCL)	71.5–75.9%	Specific scaffold designs maintained	N. r.	Not degraded over 28 days	[82]

**Table 4:** Comparative structure–properties and characteristics of representative nanoparticle classes incorporated into bio-based materials for biomedical 3D printing. The table summarizes dominant reinforcement mechanisms, rheological effects, and principal limitations that define the balance between performance enhancement and printability.

NPs Type	Dominant Reinforcement Mechanism	Rheological Impact	Principal Limitation
Metallic	Predominantly particulate reinforcement with limited stress-transfer efficiency	Moderate increase in zero-shear viscosity and elastic response	Dose-dependent cytotoxicity and aggregation tendency
Ceramic/ Inorganic	Stiffness contrast and load-bearing reinforcement	Progressive increase in viscosity and yield stress	Brittleness at elevated filler loadings
Carbon-based (sp^2^)	Percolation-driven network formation	Abrupt increase in viscoelastic properties near percolation threshold	Dispersion sensitivity and processing window narrowing
Nanodiamonds (sp^3^)	Surface-mediated interfacial reinforcement	Moderate enhancement of elastic behavior depending on dispersion	Aggregation and dispersion complexity
Polymeric	Improved mechanical load transfer and enhanced inter-layer adhesion	Increase in viscosity and shear-thinning behavior	Aggregation, nozzle clogging and poor flow and shape fidelity

## Data Availability

No new data were created or analyzed in this study.
